# Increased Tumor Necrosis Factor Superfamily Members in Neuroinflammatory Schizophrenia and Bipolar Disorder Midbrains

**DOI:** 10.1016/j.bpsgos.2025.100650

**Published:** 2025-11-01

**Authors:** Gerardo Mendez-Victoriano, Yunting Zhu, Layla Neuhaus, Suhaana Shaik, Frank Middleton, Yuji Kondo, Amir Fayyazuddin, Daniel Hoeppner, Sofía Puvogel, Astrid Alsema, Laura Kracht, Mitsuyuki Matsumoto, Bart J.L. Eggen, Adam K. Walker, Maree J. Webster, Iris E.C. Sommer, Cynthia S. Weickert

**Affiliations:** a Department of Neuroscience and Physiology, Upstate Medical University, Syracuse, New York; b Neuroscience Research Australia, Sydney, New South Wales, Australia; c School of Clinical Medicine, Faculty of Medicine and Health, University of New South Wales, Sydney, New South Wales, Australia; d Laboratory of ImmunoPsychiatry, Neuroscience Research Australia, Randwick, New South Wales, Australia; e Astellas Pharma Inc., Tokyo, Japan; f Astellas West Coast Innovation and Research Center, South San Francisco, California; g Astellas Engineered Small Molecules US, Inc., Cambridge, Massachusetts; h Cognitive Neuroscience Section, Department of Biomedical Sciences of Cells and Systems, University Medical Center Groningen, University of Groningen, Groningen, the Netherlands; i Department of Biomedical Sciences, University Medical Center Groningen, University of Groningen, Groningen, the Netherlands; j Arialys Therapeutics, Inc., La Jolla, California; k Laboratory of Brain Research, Stanley Medical Research Institute, Rockville, Maryland; l Department of Psychiatry, Brain Center Rudolf Magnus, University Medical Centre Utrecht, Utrecht, the Netherlands

**Keywords:** Bipolar disorder, Midbrain, mRNA, RNA-seq, Schizophrenia, TNFSF

## Abstract

**Background:**

Neuroinflammation is a key neuropathological finding in schizophrenia and bipolar disorder, as increased cytokines are found in the midbrain of these individuals. However, the most upregulated inflammatory cytokines and most activated downstream signaling pathway(s) are unidentified.

**Methods:**

We aimed to identify the most robust transcriptional change in the schizophrenia midbrain by bulk RNA sequencing (RNA-seq) and to confirm the cellular source and magnitude of change by single-nucleus RNA-seq, reverse transcriptase–polymerase chain reaction (RT-PCR), and immunohistochemistry in 61 healthy controls, 63 schizophrenia cases, and 33 bipolar disorder cases stratified into low- and high-inflammation groups.

**Results:**

By RNA-seq, the TNF superfamily (TNFSF) pathway messenger RNAs (mRNAs) were among the most changed in high-inflammation schizophrenia (all *p*s ≤ .01), with TNFSF receptors (*TNFR1*, *TNFR2*, and *FAS*) being most highly expressed in astrocytes and microglia. Using RT-PCR, we confirmed that 5 TNFSF receptor mRNAs (*TNFR1*, *TNFR2*, *DR4*, *FAS*, and *TWEAKR*, all *p*s ≤ .01) were increased in high-inflammation schizophrenia/bipolar disorder cases compared with low-inflammation controls. Furthermore, the means for mRNA encoding cell death–related proteins acting downstream of TNF receptors (*P53*, *CASP1*, *CASP7*, *CASP8*; all *p*s ≤ .05) were increased in high-inflammation schizophrenia, as were mRNAs encoding proteins regulating cell survival (*BCL2* and *MCL1*, all *p*s ≤ .01). All 5 TNFSF receptor mRNAs positively correlated with effector protein mRNAs (all *p*s ≤ .05) and with the astrocyte-related marker *GFAP* mRNA (all *p*s ≤ .001).

**Conclusions:**

Our results suggest that TNFSF transcripts represent the main activated inflammatory pathway in the midbrains of people with schizophrenia, which overlaps somewhat with bipolar disorder. These findings highlight the need for anti-inflammatory interventions targeting TNF/TNFSF receptors to test for therapeutic benefits in psychiatric patients displaying elevated inflammation.

## Plain Language Summary

Neuroinflammation is thought to play a key role in schizophrenia and bipolar disorder, but the most active inflammatory pathway has remained unidentified. Using RNA sequencing, confirmatory qPCR, and immunohistology, we identified the TNF superfamily as the most strongly activated inflammatory pathway in the midbrain of individuals with psychosis-related disorders with elevated inflammation. These results suggest that therapies targeting TNF/TNFSF receptors may benefit schizophrenia and bipolar disorder patients with heightened brain inflammation.

Neuroinflammation is found in multiple brain regions in people with schizophrenia and bipolar disorder (1,2). In almost half the cases with schizophrenia and one-third of cases with bipolar disorder, a 200% to 700% increase in messenger RNAs (mRNAs) encoding proinflammatory cytokines such as interleukin (IL) 1β, IL-8, IL-6, and tumor necrosis factor (TNF) have been found in the blood (3, 4, 5), subependymal zone (6), prefrontal cortex (PFC) (7,8), and midbrain (9,10). Interestingly, it has been suggested that the mammalian midbrain contains a more active (11) and higher proportion of resident immune cells than other brain regions such as the PFC (12,13), making it more likely to be responsive to inflammatory insults (11,14). This midbrain inflammatory susceptibility may contribute to the higher inflammation that has been reported in the midbrain compared with the PFC in schizophrenia and bipolar disorder subgroups with elevated inflammation (7,9,10). We previously identified *IL-6*, *IL-8*, *IL-1β*, and *SERPINA3* mRNAs as the most upregulated inflammation markers in the PFC of schizophrenia cases, whereas the most discriminatory markers in the midbrain also included *TNF* mRNA (9,10,15). However, because the survey of cytokine changes in the midbrain of individuals with schizophrenia was based on cytokines identified via RNA sequencing (RNA-seq) in the cortex, the most upregulated inflammation-related transcripts in the midbrains of schizophrenia and bipolar disorder cases have not yet been determined.

We and others have found reduced dopaminergic-related markers in the midbrain/substantia nigra (SN) of people with schizophrenia and bipolar disorder with increased proinflammatory-related markers (16, 17, 18, 19), suggesting that increased inflammation could impair dopamine function/homeostasis in the midbrain of individuals with psychosis. Dopamine imbalance is a core pathophysiological hallmark of schizophrenia and also occurs in bipolar disorder (20, 21, 22, 23, 24). However, the study of dopamine dysfunction in psychiatric disorders has been focused primarily on the PFC and striatum. The midbrain has been underevaluated, despite being the subcortical region where most of the brain’s dopamine originates (25). Recent findings have demonstrated presynaptic dopaminergic dysfunction (17,18,26), reduced inhibitory interneuron markers and GABAergic (gamma-aminobutyric acidergic) receptor mRNAs (17), and elevated glutamatergic receptor mRNAs (27) in the SN of individuals with schizophrenia. Similarly, in bipolar disorder, there are reduced tyrosine hydroxylase (TH) mRNA levels (19), increased VMAT2 signal (28), and increased anatomical volume (29, 30, 31) of the SN, thus highlighting the importance of the SN in schizophrenia and bipolar disorder pathophysiology. Furthermore, we have discovered that the increase in proinflammatory markers and gliosis is as robust in the midbrain of both psychiatric disorders as it is in cortical regions (7,9,10). Additionally, high-inflammation subgroups are prominent in the midbrain in individuals with schizophrenia (∼50%) and in those with bipolar disorder (∼30%) but are rare in control individuals (∼5%), suggesting that inflammation may be more discriminatory in this brain region (10). Therefore, we believe that discovering the transcriptional changes associated with inflammatory activation in the midbrains of people with schizophrenia and bipolar disorder could help prioritize inflammation-based biomarkers and dopaminergic-related treatments for pharmacological intervention.

Herein, we first used discovery-driven bulk RNA-seq to identify the most significantly upregulated inflammation-associated pathways in highly inflamed schizophrenia midbrains compared with low-inflammation control midbrains. We discovered that the TNF superfamily (TNFSF) was the most significantly changed cytokine pathway. Upon activation, TNFSF pathways trigger complex downstream signaling cascades associated with inflammation and programmed cell death mechanisms such as apoptosis (32). In this regard, increased expression of TNFSF receptors TNFR1/2 (TNFRSF1A/B), FAS (TNFRSF6), DR4 (TNFRSF10A), and TWEAKR (TNFRSF12A) (33, 34, 35, 36, 37, 38, 39, 40, 41) and downstream markers BCL2 (42,43), BAX (42, 43, 44), BID (42), CASP3 (43,44), CASP8 (45), and CASP9 (44) have been found in the serum, hippocampus, and PFC of people with schizophrenia and bipolar disorder. However, these TNFSF targets have not been measured in the midbrains of people with schizophrenia and bipolar disorder compared with control individuals, and whether their expression could be changed by inflammatory status is unknown.

To corroborate the bulk RNA-seq findings, we evaluated mRNA levels in a larger schizophrenia cohort by reverse transcriptase–polymerase chain reaction (RT-PCR). Additionally, as we have found similar neuropathological features in both schizophrenia and bipolar disorder groups with an elevated inflammatory signature [increased macrophage expression, reduced *TH* mRNA levels (19), and increased expression of angiogenic transcripts (46)], and both psychiatric disorders share inflammatory risk-related genes (47), we included a bipolar disorder group in the analyses. Therefore, we evaluated the mRNA levels of TNFSF receptors and downstream members in schizophrenia and bipolar disorder midbrains compared with control midbrains and schizophrenia/bipolar disorder cases stratified by low- and high-inflammation compared with low-inflammation controls. Lastly, we used single-nucleus RNA-seq (snRNA-seq) and immunohistochemistry to identify the brain cell type expressing TNF receptors (TNFRs) in the human midbrain.

## Methods and Materials

### Midbrain Cohort Demographics

Human postmortem midbrain tissue was obtained from the New South Wales Brain Tissue Resource Centre and Stanley Medical Research Institute Array cohorts. The whole mRNA cohort included 61 healthy controls, 63 schizophrenia cases, and 33 bipolar disorder cases (Table 1). Age, postmortem interval (PMI), and RNA integrity number (RIN) did not differ significantly between diagnostic groups. The bipolar disorder and schizophrenia groups had lower brain pH than the control group (both *p*s ≤ .001). Consistent with clinical observations, the bipolar disorder group had a lower average lifetime chlorpromazine equivalent dose (*p* = .001) and fewer years with illness (*p* = .05) compared with the schizophrenia group. Our study was approved by the Human Research Ethics Committee at the University of New South Wales (#HREC: HC230253). All cases were previously categorized into low- or high-inflammation subgroups using a 2-step recursive clustering analysis based on mRNA expression of *SERPINA3*, *IL-1β*, *IL-6*, and *TNF-α* (9,10). Cases with any underlying inflammation-related condition were excluded from the study. Cases for the RNA-seq and immunohistochemistry cohorts (Tables S1–S3) are a subset of the whole mRNA cohort. More details on midbrain cohort demographics, RNA extraction, complementary DNA synthesis (cDNA), quantitative PCR (qPCR), bulk RNA-seq and snRNA-seq, and FAS immunostaining are provided in the Supplement. These methods are summarized below.

**Table 1:** Demographic Characteristics of the Whole Messenger RNA Cohort

Demographics	Control, *n* = 61	Schizophrenia, *n* = 63	Bipolar Disorder, *n* = 33	Statistics
Age, Years	47.42 ± 9.92 [22–67]	46.49 ± 10.81 [19–67]	45.42 ± 10.83 [19–64]	*F*_2,154_ = 0.40, *p* = .671
Sex, Female:Male	16:45	18:45	18:15	χ^2^_2_ = 8.73, *p* = .013
Brain pH	6.63 ± 0.26	6.49 ± 0.22	6.43 ± 0.30	*H*_2,157_ = 17.41, *p* < .001
PMI, Hours	30.88 ± 12.81	32.93 ± 16.52	37.96 ± 18.90	*H*_2,157_ = 1.91, *p* = .384
RIN	5.81 ± 1.20	6.00 ± 1.29	6.30 ± 1.24	*H*_2,157_ = 3.89, *p* = .143
Brain Weight, g	1445.42 ± 152.08	1416 ± 143.10	1397.57 ± 142.33	*F*_2,111_ = 0.91, *p* = .405
Suicide as Cause of Death, Yes:No^a^	–	14:49	15:18	χ^2^_1_ = 5.54, *p* = .019^b^
Age at Onset, Years^a^	–	21.34 ± 6.21	25.53 ± 9.36	*U*_95_ = 757, *p* = .048^b^
Duration of Illness, Years^a^	–	24.65 ± 11.90	19.87 ± 9.64	*t*_94_ = 1.98, *p* = .05^b^
Lifetime Chlorpromazine Equivalent Dose, mg^a^	–	5,786,826.526 ± 6,897,882.835	495,757.576 ± 1,157,944.786	*U*_90_ = 151, *p* < .001^b^

Values are presented as mean ± SD or *n*.

PMI, postmortem interval; RIN, RNA integrity number.

a Statistical analysis between psychiatric cases only.

b Statistical significance.

### RNA Extraction

Total RNA was extracted from fresh-frozen ventral midbrain tissue using the TRIzol (#15596026; Invitrogen) extraction method, as previously described (48). RNA concentration (by NanoDrop) and quality were determined (Agilent Technologies 2100 Bioanalyzer).

### Midbrain Bulk RNA-Seq and Analysis

The bulk RNA-seq included 20 control and 40 schizophrenia cases (Table S1). An average of 296 million total reads and 153 million unique paired-end reads were attained per sample, with the average Phred quality score >34 across all samples and positions before and after trimming. Gene counts were analyzed for differential expression across groups after filtering low-expressed genes using DESeq2 (adjusted *p* < .05 using Benjamini-Hochberg adjustment).

### cDNA Synthesis and qPCR

cDNA synthesis was performed from 1 μg total RNA per case using the SuperScript III First-Strand Synthesis kit. mRNA expression of the TNFSF markers was measured by RT-qPCR using the Fluidigm BioMarkTM HD system with predesigned TaqMan Gene Expression Assays (Thermo Fisher Scientific) (Table S2) and calculated by normalizing the concentration of the target transcript to the geometric mean of 3 housekeeper transcripts: *GAPDH* (Hs99999905_m1), β-glucuronidase (Hs99999908_m1), and ubiquitin C (Hs00824723_m1). There were no statistically significant differences in the geometric mean of the housekeeper genes between diagnostic groups (*H*_2,157_ = 4.94, *p* = .084) (Figure S1).

### Midbrain snRNA-Seq Analyses

Human postmortem samples, nuclei isolation, and snRNA-seq steps were detailed previously (49,50). The snRNA-seq included 14 control and 20 schizophrenia cases (Table S3). Single-nucleus cDNA libraries were constructed using the 10X Genomics Chromium Single Cell 3′ Reagents Kit v3 according to the user guide. The mean sequencing depth was 223 million reads per sample, and the mean number of reads per nucleus was 43,263. The mean estimated number of nuclei was 5184 per sample, and the mean total detected transcripts was 38,326 per sample (median unique molecular identifier/nuclei = 2780). CellRanger’s filtered feature_bc_matrix output was used to analyze the data. The integrated Seurat object was used to run unsupervised clustering analyses using FindNeighbors (dims = 1:30, k.param = 10) and FindClusters (resolution = 0.4) functions to identify cell types/clusters based on marker genes. *FAS*, *TNFR1*, and *TNFR2* gene expression levels were visualized using Uniform Manifold Approximation and Projection plots. The expression of TNFSF receptors *DR4* and *TWEAKR* was deemed too low to be accurately mapped to cell clusters.

### FAS Immunostaining

Briefly, fresh-frozen midbrain sections (14 μm) (control *n* = 9, schizophrenia *n* = 18, and bipolar disorder *n* = 18) (Table S4) were used for brightfield immunohistochemistry to detect FAS+ cells. Slides were incubated (4 °C overnight) with primary antibody (mouse anti-FAS, 1:250; sc-8009) washed in 1× phosphate-buffered saline (PBS), and the next day, they were incubated with a secondary antibody (goat anti-mouse IgG biotinylated, 1:500; BA-9200; Vector Laboratories), before being washed and incubated with avidin-biotin-peroxidase complex (PK-4000; VectaStain; Vector Laboratories) followed by PBS washes and visualization with a DAB reaction. FAS+ cells in the SN pars compacta were defined as positive when DAB staining colocalized with nuclear thionin staining, and both were found in the same focal plane. Representative FAS+ cell microphotographs were taken in the SN, cerebral peduncles, and superior colliculi at 40× with a Nikon Eclipse 80i microscope (Nikon Instruments Inc.).

### Statistical Analysis

For analyses between groups, analysis of variance (ANOVA)/analysis of covariance (ANCOVA) + least significant difference post hoc tests were used on the normally distributed continuous variables, whereas the Kruskal-Wallis + Dunn-Bonferroni post hoc test or Quade’s ANCOVA were used on the non-normally distributed continuous variables. All between-group tests were corrected for multiple comparisons across groups. Categorical variables were tested with Pearson’s χ^2^ test. Correlation analyses were performed using the parametric Pearson’s (*r*) and nonparametric Spearman’s rank (ρ) correlations for normally and non-normally distributed variables, respectively. Demographic variables (age, RIN, PMI) with significant correlations/associations with dependent variables were used as covariates (Tables S5 and S6). Brain pH was not included as a covariate because it is lower in postmortem samples of schizophrenia brains (51), and acidosis is typical of inflamed tissue (52). For completeness, results including brain pH as a covariate are included in the Supplement (Table S7). There were no significant differences in our dependent variables by sex (Table S8). Statistical analyses were performed using SPSS Statistics (version 25; IBM Corp.). Data graphs were plotted using means ± SEMs using GraphPad Prism (version 8.0.1) software.

## Results

### TNF and Apoptosis Are Among the Most Upregulated Pathways in High-Inflammation Schizophrenia Via Bulk RNA-Seq

**Figure 1:**
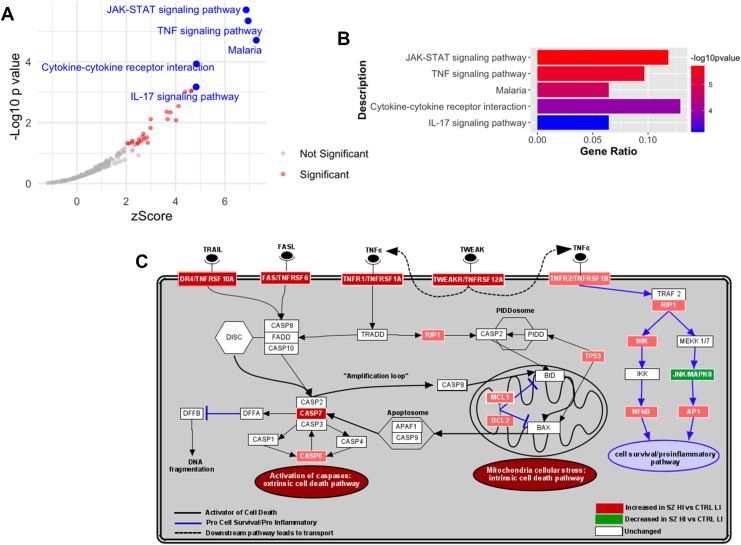
Bulk RNA-seq results of the TNF pathway. **(A, B)** Bulk RNA-seq volcano **(A)** and bar **(B)** plots of the most upregulated mRNA pathways in the HI SZ group compared with the LI CTRL group. **(C)** Summary diagram of the apoptosis-related TNF pathway messenger RNAs changed significantly in the HI SZ group compared with the LI CTRL group. Darker colors represent a higher magnitude of change. CTRL, control; HI, high-inflammation; LI, low-inflammation; mRNA, messenger RNA; RNA-seq, RNA sequencing; SZ, schizophrenia; TNF, tumor necrosis factor.

We found 250 differentially expressed (DE) genes between control and schizophrenia samples (Table S8). The Kyoto Encyclopedia of Genes and Genomes enrichment pathway analyses identified multiple inflammation-related pathways upregulated in schizophrenia cases compared with controls, including inflammatory cytokine interaction pathways. The upregulated pathways with the highest magnitude of change were JAK-STAT and TNF signaling pathways (all *p*s < .001) (Figure 1A, B). DE downstream members of the TNF pathway were also part of the apoptosis pathway (Figure 1C and Figure S2).

### TNFSF Pathway Member Transcripts Are Changed Via Differential Expression From Bulk RNA-Seq

We found multiple TNFSF receptor transcripts increased in high-inflammation schizophrenia cases compared with low-inflammation controls, including *TNFRSF1A* (log_2_ fold change [FC] = 0.91, adjusted *p* [*p* adj.] = 1.93 × 10^−10^, +87.44% increase), *TNFRSF10A* (log_2_FC = 0.56, *p* adj. = 1.70 × 10^−3^, +47.88%), *TNFRSF10B* (log_2_FC = 0.43, *p* adj. = .05, +35.08%), and *FAS* (log_2_FC = 0.85, *p* adj. = 3.67 × 10^−6^, +79.75%) (Figure 1C). Transcripts shared with the apoptosis pathway were also increased, including *MCL1* (log_2_FC = 1.27, *p* adj. = 2.58 × 10^−9^, +141.99%), *BCL2A1* (log_2_FC = 2.68, *p* adj. = 1.16 × 10^−8^, +540.89%), and *CASP7* (log_2_FC = 0.65, *p* = 1.42 × 10^−3^, +56.89%) (Figure 1C). Conversely, other TNFSF downstream mRNAs were unchanged (*BAX*, *BID*, *CASP1*, *CASP3*, *CASP8*, and *CASP9*) in high-inflammation schizophrenia cases compared with low-inflammation controls. As enrichment analyses indicated collective dysregulation of TNF pathway members, we analyzed TNFSF receptors and downstream transcripts using RT-PCR. This approach provides higher detection sensitivity and greater statistical power by enabling analysis of a larger sample size, allowing robust testing for changes in transcripts encoding proteins involved in inflammation and cell death. To confirm and compare transcriptional changes in individual TNFSF members and to extend these findings to bipolar disorder, we measured TNFSF mRNA levels in a larger cohort using RT-PCR (Table 1).

### TNFSF Receptor Transcripts Are Increased in Schizophrenia and Bipolar Disorder Via RT-PCR

When analyzed by diagnosis, we found that compared with the control group, the schizophrenia group had significantly higher mRNA levels of all 5 *TNFSF* receptors examined: *FAS* (*F*_2,145_ = 9.46, *p* ≤ .001), *TNFR1* (*H*_2_ = 13.30, *p* = .001), *TNFR2* (*F*_2,146_ = 6.91, *p* ≤ .001), *TWEAKR* (*H*_2_ = 15.08, *p* = .001), and *DR4* (*H*_2_ = 18.02, *p* ≤ .001) (all *p* adj. ≤ .05) (↑16% [average percentage change]). Similarly, the bipolar disorder group had significantly higher mRNA levels of *TNFR1* and *TWEAKR* (all *p* adj. ≤ .05) (Figure S3).

### TNFSF Receptor Transcripts Are Increased in High-Inflammation Schizophrenia and High-Inflammation Bipolar Disorder

**Figure 2:**
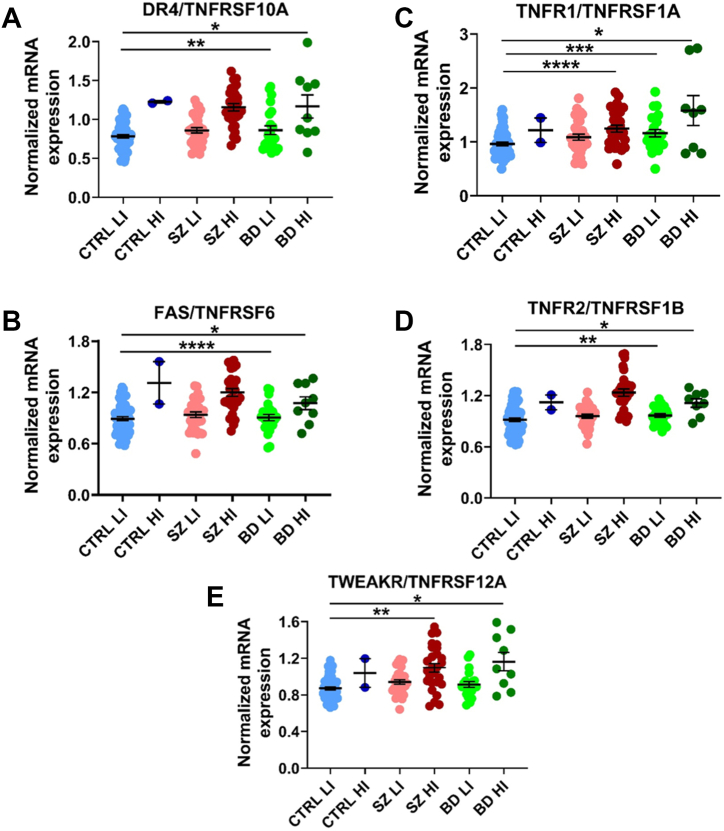
mRNA expression of TNFSF receptors. **(A–E)** Normalized mRNA expression levels of the TNFSF receptors *DR4***(A)**, *FAS***(B)**, *TNFR1***(C)**, *TNFR2***(D)**, and *TWEAKR***(E)**. Data points are individual cases. ∗*p* ≤ .05, ∗∗*p* ≤ .01, ∗∗∗*p* ≤ .001, ∗∗∗∗*p* ≤ .0001. BD, bipolar disorder; CTRL, control; HI, high-inflammation; LI, low-inflammation; mRNA, messenger RNA; SZ, schizophrenia; TNFSF, tumor necrosis factor superfamily.

We found that compared with the low-inflammation control group, both high-inflammation schizophrenia and high-inflammation bipolar disorder subgroups had significantly higher mRNA levels of all 5 *TNFSF* receptors examined, *FAS* (*F*_4,142_ = 11.10, *p* ≤ .001), *TNFR1* (*F*_4,143_ = 6.66, *p* ≤ .001), *TNFR2* (*H*_4_ = 42.72, *p* ≤ .001), *TWEAKR* (*H*_4_ = 24.51, *p* ≤ .001), and *DR4* (*H*_4_ = 38.72, *p* ≤ .001) (all over ↑35%, all *p* adj. ≤ .05) (Figure 2A–E). Additionally, *TNFR1* mRNA was increased in the low-inflammation bipolar group compared with low-inflammation control group (↑16%, *p* adj. ≤ .05). Lastly, *FAS*, *DR4*, and *TNFR2* mRNAs were increased in high-inflammation schizophrenia compared with low-inflammation bipolar/schizophrenia groups (↑29%, all *p* adj. ≤ .01).

### Many TNFSF Downstream Pathway Transcripts Are Changed in High-Inflammation Schizophrenia and/or Bipolar Disorder

**Figure 3:**
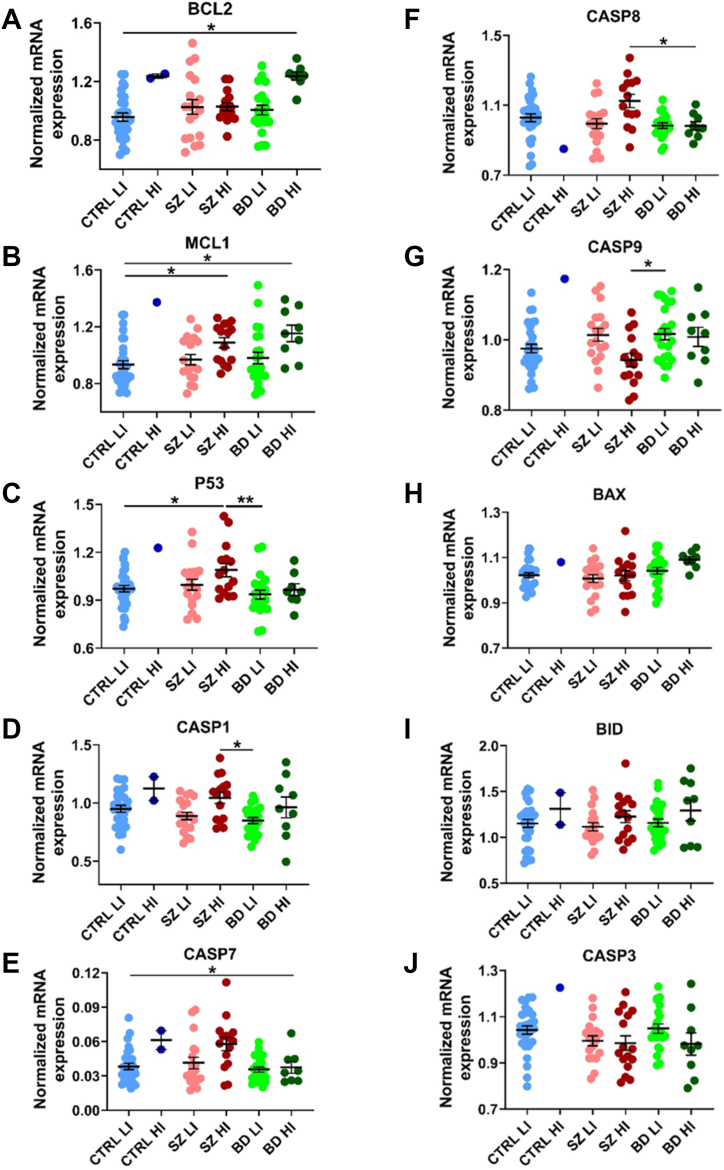
mRNA expression of TNFSF pathway downstream markers. **(A–J)** Normalized mRNA expression levels of the cell death–associated TNFSF downstream pathway markers *BCL2***(A)**, *MCL1***(B)**, *P53***(C)**, *CASP1***(D)**, *CASP7***(E)**, *CASP8***(F)**, *CASP9***(G)**, *BAX***(H)**, *BID***(I)**, and *CASP3***(J)**. Data points are individual cases. ∗*p* ≤ .05, ∗∗*p* ≤ .01. BD, bipolar disorder; CTRL, control; HI, high-inflammation; LI, low-inflammation; mRNA, messenger RNA; SZ, schizophrenia; TNFSF, tumor necrosis factor superfamily.

*BCL2* mRNA levels were increased only in the high-inflammation bipolar disorder subgroup (*F*_4,88_ = 4.52, *p* ≤ .01) compared with all other subgroups (all *p* adj. ≤ .01) (Figure 3A). *MCL1* (*H*_4_ = 17.31, *p* = .002) and *P53* (*F*_4,88_ = 2.85, *p* = .028) mRNAs were increased in high-inflammation schizophrenia (*MCL1* and *P53*) and high-inflammation bipolar disorder (*MCL1*) subgroups compared with the low-inflammation control group (all *p* adj. ≤ .05) (Figure 3B, C). *CASP1* (*F*_4,32.47_ = 3.79, *p* = .012), *CASP8* (*F*_4,34.21_ = 3.30, *p* = .022) and *P53* mRNAs were increased in high-inflammation schizophrenia compared with low- (*CASP1* and *P53*) and high- (*CASP1* and *CASP8*) inflammation bipolar subgroups (all *p* adj. ≤ .05) (Figure 3C, D, F), while *CASP7* (*F*_4,89_ = 3.72, *p* = .008) mRNA was increased in the high-inflammation schizophrenia subgroup compared with all other subgroups (all *p* adj. ≤ .01) (Figure 3E). Conversely, *CASP9* mRNA was decreased in the high-inflammation schizophrenia subgroup (*F*_4,89_ = 3.34, *p* = .013) compared with the low-inflammation bipolar disorder subgroup (*p* adj. ≤ .01) (Figure 3G). We did not detect differences in mRNA levels of the remaining effectors (*BAX*, *BID*, and *CASP3*) across diagnostic/inflammation subgroups (Figure 3H–J).

### Positive Correlations Are Found Between TNFSF Receptor Transcripts and Their Downstream Pathway Transcripts

We discovered that all 5 inflammation/cell death–associated *TNFSF* receptor mRNAs had positive associations with downstream signaling pathway mRNA markers (all *p*s ≤ .05), with *FAS* and *TNFR1* having the strongest and most numerous (8/10) and significant correlations (Table S9).

### TNFSF Receptors FAS, TNFR1, and TNFR2 Are Mostly Expressed by Glial Cells, With TNFR1 and FAS Being More Expressed in Astrocytes in High-Inflammation Schizophrenia

**Figure 4:**
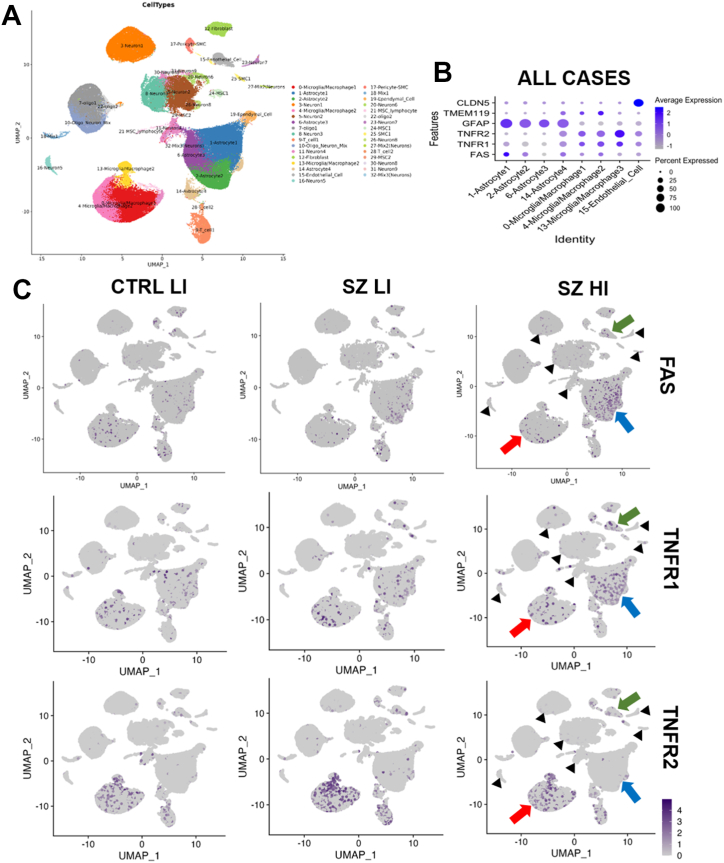
Single-nucleus RNA sequencing plots of TNFSF receptor markers across cell-type clusters. **(A)** UMAP plot with colors representing cell-type clusters. **(B)** Dot plots of percentage and average expression of the TNFSF receptors *FAS*, *TNFR1*, and *TNFR2* in astrocytes, microglia/macrophages, and endothelial cell clusters across in all cases. **(C)** UMAP plots of TNFSF receptors *FAS*, *TNFR1*, and *TNFR2* between CTRL LI, SZ LI, and SZ HI groups. Red arrows, macrophage/microglia; blue arrows, astrocytes; green arrows, endothelial cells; black arrows, neuron clusters. CTRL, control; HI, high-inflammation; LI, low-inflammation; SZ, schizophrenia; TNFSF, tumor necrosis factor superfamily; UMAP, Uniform Manifold Approximation and Projection.

The cellular source and distribution of TNFSF receptors were examined by cell-clustering analysis from snRNA-seq data from our previous studies (49,53). We identified 32 neural cell type clusters, 4 of which were astrocyte-related and 3 of which were microglial/macrophage-related clusters (Figure 4A). We found that *FAS* mRNA was mostly expressed by astrocytes, whereas *TNFR1* and *TNFR2* mRNAs were mostly found in microglia/macrophages (followed by astrocytes), with low expression of all 3 TNFRs in endothelial cells (Figure 4B). Notably, *FAS* and *TNFR1* appeared to be more highly expressed in astrocytes in high-inflammation schizophrenia compared with the low-inflammation subgroups (Figure 4C, blue arrows). *TNFR2* appeared higher in microglia/macrophages than in astrocytes in all subgroups (Figure 4C, red arrows). None of the 3 TNFR transcripts appeared significantly expressed in neuron clusters across subgroups (Figure 4C, black arrowheads).

### FAS+ Cells Have Astrocyte-Like Morphologies

**Figure 5:**
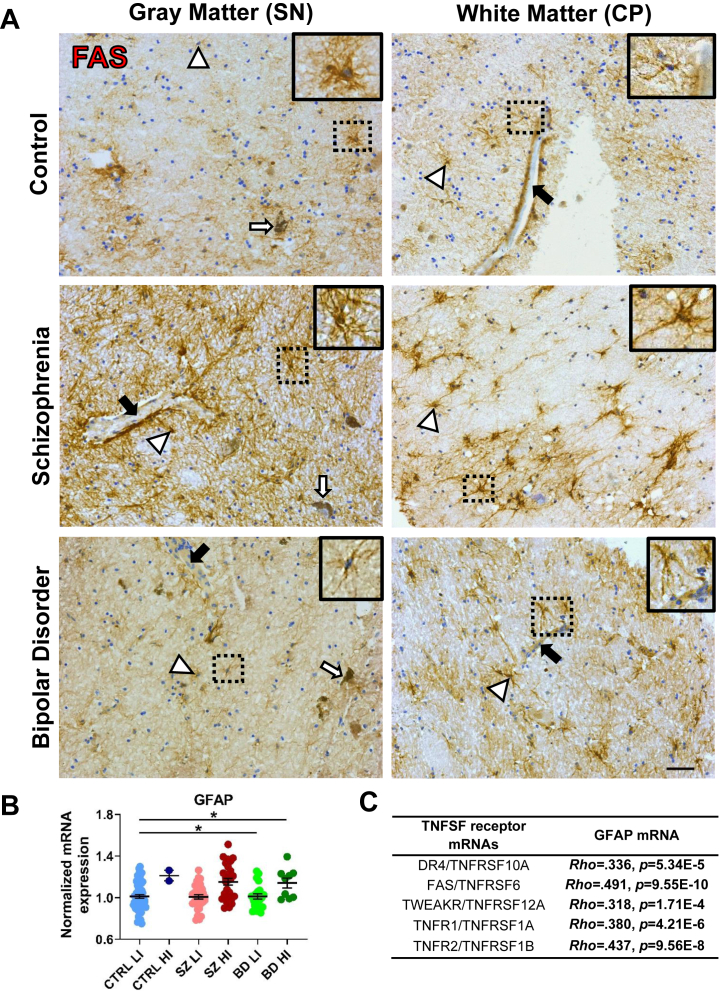
Expression of FAS+ cells and *GFAP* mRNA. **(A)** Location and morphology of cells immunostained with the TNFSF receptor FAS marker. Scale bar = 50 μm. White arrowheads and insets, ramified-like FAS+ cells; black arrows, blood vessels; white arrows, neuromelanin+ cells. **(B)** Normalized mRNA levels of the astrocyte marker *GFAP*. Data points are individual cases. **(C)** Positive correlations between the mRNA expression of *GFAP* and TNFSF receptors *DR4*, *FAS*, *TWEAKR*, and *TNFR1*/*TNFR2*. ∗*p* ≤ .05. BD, bipolar disorder; CP, cerebral peduncle; CTRL, control; HI, high-inflammation; LI, low-inflammation; mRNA, messenger RNA; SN, substantia nigra; SZ, schizophrenia; TNFSF, tumor necrosis factor superfamily.

We confirmed the protein expression of FAS in astrocytes by immunohistochemistry. The FAS immunostaining was located in midbrain subregions in a patchy pattern in both gray and white matter. FAS+ cells had ramified processes (Figure 5A, white arrowheads and insets), and FAS+ immunoreactivity often surrounded blood vessels (Figure 5A, black arrows), suggesting astrocyte-like profiles. FAS+ cells were also found in the dorsal midbrain, close to the central aqueduct and the superior colliculi (Figure S4). FAS+ cells were found in 2/9 controls (22.2%), 7/18 schizophrenia cases (38.9%), and 4/18 bipolar disorder cases (22.2%), with no significant change according to diagnosis (χ^2^_2_ = 1.46, *p* = .482). Because immunoreactivity was not detected in all cases, quantification of the FAS protein was not undertaken.

### mRNA Levels of GFAP Are Increased in High-Inflammation Schizophrenia and Bipolar Disorder and Positively Associated With TNFSF Receptor Transcripts

Since *FAS* and *TNFR1* mRNAs were highly expressed in astrocytes in schizophrenia by snRNA-seq, we measured a marker of reactive astrocytes, *GFAP* mRNA, to determine whether these changes could be part of a generalized astrocyte response to inflammation. We found that *GFAP* mRNA levels were increased in high-inflammation schizophrenia (*F*_4,138_ = 5.08, *p* = .001) compared with all low-inflammation subgroups and in high-inflammation bipolar disorder cases compared with low-inflammation controls (all *p* adj. ≤ .05) (Figure 5B). Additionally, *GFAP* mRNA had strong positive correlations with all 5 *TNFSF* receptor mRNAs (all *p*s ≤ .01) (Figure 5C).

## Discussion

Overall, we found robust evidence for *TNFSF* mRNAs to be part of the major activated inflammatory pathways in schizophrenia midbrain. The activation of TNFRs was not unique to schizophrenia, as changes in this pathway were also found in bipolar disorder. These results were mainly evident in subsets of cases with schizophrenia (∼50%) and bipolar disorder (∼30%) that displayed elevated inflammation, as transcriptional expression in the low-inflammation psychiatric groups was often similar to that of the healthy control group. We discovered that midbrain astrocytes and microglia/macrophages expressed *TNFSF* receptors *FAS*, *TNFR1*, and *TNFR2* at low levels in controls and at high levels in schizophrenia cases. As we found molecular mRNA changes downstream of TNFSF receptors, our results show that increased brain TNF and TNFR have an impact on cellular response pathways in the human midbrain.

### Increased TNFSF mRNAs Indicate Activation of Inflammatory and Cell Death–Related Pathways

Our results indicate increased inflammation and potential apoptotic-related activation in the midbrains of inflamed patients with psychosis through increased TNFSF receptor synthesis and suggest that glial cells are poised to respond to increased TNF directly. Members of the TNFSF pathway are part of cell death pathways like apoptosis/pyroptosis (32,54), and while it has been debated as to whether cell death occurs in the brains of people with schizophrenia/bipolar disorder, increased TNF can directly damage neural cells in several neuroinflammatory conditions (55, 56, 57) and could result in altered neuronal function. *TNF* and its receptor TNFR1, have been suggested as plausible biomarkers for the prognosis and progression of schizophrenia and bipolar disorder, as increased soluble TNFR1 (sTNFR1) serum levels are found in acute patients (34,58); sTNFR1 is increased in treatment-resistant patients compared with nonresistant patients and healthy control participants (59). Additionally, elevated sTNFR1 and TNF have been associated with worse clinical functioning in individuals with schizophrenia (59,60) and bipolar disorder (61). Clinical trials with anti-TNF–related biological adjuvants like adalimumab (62) and penfluridol (63,64) reported improvement of general symptoms in patients with schizophrenia. Furthermore, the improvement effect of anti-TNF drugs on mood-related symptoms indicates that TNF may be a plausible target for the treatment of psychiatric-related disorders (65,66). These findings highlight that TNF and its receptors could be plausible inflammation-based targets for pharmacological treatment, specifically for schizophrenia and bipolar disorder cases with elevated inflammation.

### TNFSF Receptor mRNAs Are Expressed by Glia

The increased expression of *FAS* and *TNFR1* in astrocytes, along with increased *GFAP* mRNA expression and positive correlations between TNFRs and *GFAP* mRNAs in high-inflammation schizophrenia, suggests that reactive astrocytes could be the main source of *FAS*/*TNFR1* in the inflamed midbrain. Astrocytes are resistant to Fas-mediated cell death (67,68). In contrast, *FAS* ligation on astrocytes produces proinflammatory activation of chemokine (CC-motif) ligand 2, IL-6, and IL-8 (67,68), all of which are known to be increased in the schizophrenia brain (3,4,9,10,15,69,70). Based on the above, it has been proposed that FAS-expressing, cell death–resistant astrocytes induce proinflammatory cytokine/chemokine release upon FAS/L binding, which can promote cell death of surrounding neurons and oligodendrocytes, potentially leading to neurodegeneration/demyelination (71). Although expression of *TNFR1* in astrocytes has not been found in other central nervous system (CNS) disorders, astrocytes express *FAS* in inflammatory-related neurodegenerative diseases such as Alzheimer’s disease (72), Parkinson’s disease (73), Huntington’s disease (73), and autoimmune encephalitis (74), similar to what we found here in schizophrenia and bipolar disorder. Further studies are needed to identify which cell types are sensitive to potential FAS-induced cell death signaling and which astrocyte cytokines are produced in response to TNF.

### Increase in TNFSF Inflammatory/Pro–Cell Death–Related Receptors and Downstream mRNA Markers in Schizophrenia and Bipolar Disorder

Because we found higher levels of TNFSF markers in high-inflammation schizophrenia than in high-inflammation bipolar disorder, we suggest that TNFSF pathways could be activated more robustly in schizophrenia than in bipolar disorder under high-inflammatory states. However, reports have shown shared cell death–related transcriptional expression between schizophrenia and bipolar disorder (75), including increased levels of *TNFR1*, *TWEAKR*, and *FAS* in serum (36,58,59,76, 77, 78), the frontal cortex (39,40,79), and the hippocampus (42). Conversely, decreased serum TNFR1 levels have also been found, but mostly in early-stage patients with schizophrenia (80,81). In addition, one of the few studies that explored cell death–related markers in (hippocampus) schizophrenia and bipolar disorder found a lower percentage of inflammation and cell death–related mRNA markers significantly changed (18/44 [40%]) (42) compared with our study in the midbrain (13/17 [76%]), where we stratified by inflammation. These findings suggest that activation of cell death–associated markers in individuals with schizophrenia and bipolar disorder varies by brain region and is significantly increased in inflammatory environments.

The role of TWEAKR (TNFRSF12A) has not been widely explored in psychiatric disorders, and to our knowledge, this is the first study to find significantly increased *DR4* (*TNFRSF10A*) mRNA in high-inflammation schizophrenia and bipolar postmortem brains. *DR4* overexpression triggers apoptosis of immune cells (82). Similarly, TWEAK expression on astrocytes leads to motor neuron death via its interaction with CD163 [an inflammation-related macrophage marker (83)] and not when binding to TWEAKR (84). In this regard, we have evidence that elevated CD163 transcripts, protein, and cell densities are some of the most robust and specific changes in high-inflammation schizophrenia in multiple brain regions, including the dorsolateral PFC (69,70,85), midbrain (19,86), and subependymal zone (6,85,87). Further studies evaluating the relationship between TWEAK/R with inflammatory and neurodegenerative mechanisms in psychiatric disorders are encouraged to address the role of these cell death ligands/receptors in the neuropathological features of the brains of schizophrenia and bipolar disorder.

We found that transcripts encoding factors downstream of TNFRs were increased in high-inflammation schizophrenia, including *BCL2*, *MCL1*, *CASP7*, and *P53* mRNAs, while *BAX*, *BID*, *CASP1*, *CASP3*, *CASP8*, and *CASP9* mRNAs seemed to be unchanged by diagnosis alone. The increased levels of the anti-apoptotic/pro–cell survival markers *MCL1* and *BCL2* and unchanged levels of the pro–cell death markers *BID* and *BAX* suggest a balance toward activated cell survival mechanisms in the midbrains of individuals with schizophrenia and bipolar disorder. This may explain why cell death has been difficult to detect in the postmortem brains of people with schizophrenia or bipolar disorder. Increased protein and mRNA levels of *BAX* relative to *BCL2* have been found in the temporal cortex (88,89) and PFC (44) of patients with schizophrenia. Unchanged *CASP9* mRNA results could be due to the increased *P53* mRNA, as CASP9 suppression offers cells an escape from apoptosis when cytoplasmic P53 locally accumulates (90). However, we cannot confirm that this occurred as we have not measured protein levels or determined their subcellular distribution. Similar to our findings, unchanged *CASP3* mRNA has been shown in the hippocampus of patients with schizophrenia and bipolar disorder (42). Although *CASP3* activation is a widely used marker indicative of cell death in neurodegenerative disorders (91), activation of the intrinsic proapoptotic marker *BAX* in the absence of *CASP3* activation has been found in the temporal cortex of people with schizophrenia (90). As we and others found increased mRNA levels of multiple other cell death–related markers without the activation of *CASP3*, we suggest that, if it occurs, cell death in the midbrains of highly inflamed psychiatric individuals could be triggered via alternative markers such as *CASP7*, which we found significantly increased, and/or via a *CASP3*-independent pyroptotic path (32).

Altogether, we suggest that the change in expression of TNFSF transcripts may vary depending on the degree of inflammation, stage of disease, location (peripheral/systemic [blood] vs. CNS [brain]), and brain region being evaluated (36,61,92). However, the midbrain may be especially susceptible to TNFSF glial-based activation, as *TNF* expression is higher in the midbrain (93) and is produced by microglia/macrophages (94). As proinflammatory cytokines (95) and chemokines (96, 97) are associated with activating *TNFSF* pathways and because approximately 50% of patients with schizophrenia, 30% of patients with bipolar disorder, and 6% of healthy control participants have a highly inflamed midbrain profile when cytokines are assayed at one time point (10), we suggest that TNFSF markers could be plausible, reliable biomarkers for inflammatory disease severity in patients with schizophrenia and bipolar disorder. As TNF receptors are linked to cell death and inflammation, further studies evaluating the relationship between TNFSF pathway activation with inflammatory and degenerative events in the midbrain and other brain regions, and during different disease stages and inflammatory states, would allow us to better understand the spatiotemporal expression of these molecules and their role in schizophrenia and bipolar disorder neuropathology.

### Limitations

By discovery-driven analysis, we found increased TNFSF pathway activation, confirming that changes downstream of elevated TNF occur. However, our study has limitations. Despite confounders like use of alcohol, drugs, and smoking at the time of death did not differ statistically between inflammatory subgroups (10), we were not able to rule out the impact of antipsychotic medication in the psychiatric groups. We previously found antipsychotics to be correlated with inflammatory markers in the midbrain of individuals with schizophrenia (9,10,69,86). Herein, we found positive correlations between lifetime antipsychotic medication and mRNA expression of 3 TNFSF receptors, *DR4*, *FAS*, and *TNFR2*, and with *GFAP* (all *p*s ≤ .01) (Table S10) and 4 downstream pathway markers, *CASP7*, *CASP8*, *MCL1*, and *TP53* (all *p*s ≤ .05) (Table S11). This suggests that mRNA expression of some TNFSF factors in psychiatric cases is partially affected by long-term use of antipsychotics, or that cases with severe symptoms, and in turn, individuals needing higher levels of antipsychotics, are patients with increased midbrain TNFSF gene expression. Another limitation is the lack of analysis of the TNFSF markers at the protein level to compare/corroborate our transcriptional findings. We performed immunohistochemistry using primary antibodies against TNFR1, TNFR2, and TWEAKR; however, none provided a reliable, clear immunostaining signal (data not shown). We have previously found increased *TNF-α* mRNA levels in the midbrain of individuals with schizophrenia that were not reflected at the protein level (10), suggesting increased proteolysis of TNF-α or altered expression of transmembrane/soluble TNF-α due to inflammation (79).

A final limitation is that we included the *NeuN* marker when sorting the neuron cluster in the snRNA-seq isolation; however, it is known that *NeuN* expression is low-to-not expressed in some human midbrain neurons (98,99). Therefore, we cannot confirm that all neuron types were included in the cell clusters evaluated or that TNFSF markers are not abundantly expressed in other midbrain neuron types not captured here.

### Conclusions

Our results suggest robust midbrain immune glial cell–based transcriptional activation of TNFSF inflammation/cell death–related pathways in half the patients with schizophrenia and one-third of patients with bipolar disorder who exhibit elevated inflammation. These findings provide insight into the role of *TNF* activation in the brains of people with schizophrenia and bipolar disorder and suggest that both astrocytes and microglia are involved in the midbrain dysfunction present in these psychiatric disorders. Our findings highlight the need for novel adjuvant anti-inflammatory interventions targeting proinflammatory TNF or glial TNF receptors, such as TNFR1 and FAS, to test for potential therapeutic benefits, specifically in psychiatric patients who display elevated inflammation (100,101).

## Supplementary Material

Supplemental Methods, Figures S1–S4, and Tables S1–S12

Key Resources Table
