# Supplementary material for: Increased Tumor Necrosis Factor Superfamily Members in Neuroinflammatory Schizophrenia and Bipolar Disorder Midbrains
Source: Biol Psychiatry Glob Open Sci. 2025 Nov 1;6(2):100650. doi: 10.1016/j.bpsgos.2025.100650 (PMC12818133; doi:10.1016/j.bpsgos.2025.100650)
Supplement: Supplemental Methods, Figures S1–S4, and Tables S1–S12 [file mmc1.pdf]

SUPPLEMENTARY INFORMATION

Increased Tumor Necrosis Factor Superfamily Members in Neuroinflammatory Schizophrenia and Bipolar Disorder Midbrains

Mendez-Victoriano *et al.*

Table of Contents

Methods Supplementary

Tables Supplementary

Table Supplementary 1. Demographics of the bulk RNA-seq cohort

Table Supplementary 2. Taqman Gene Expression Assays used

Table Supplementary 3. Demographics of the snRNA-seq cohort

Table Supplementary 4. Demographics of the Immunohistochemistry cohort

Table Supplementary 5. Correlations between demographic and dependent variables in the whole mRNA cohort

Table Supplementary 6. Correlations between demographic and dependent variables in the SMRI mRNA cohort

Table Supplementary 7. Comparisons of our dependent variables when including brain pH as a covariate

Table Supplementary 8. Comparisons of our dependent variables by sex

Table Supplementary 9. Differentially expressed genes between schizophrenia and control by bulk RNA-seq

Table Supplementary 10. Positive correlations between TNFSF receptors and their downstream signaling pathway mRNA markers

Table Supplementary 11. Correlations between clinical and dependent variables in the psychiatric groups of the whole mRNA cohort

Table Supplementary 12. Correlations between clinical and dependent variables in the psychiatric groups of the SMRI mRNA cohort

Figures Supplementary

Figure Supplementary 1. Geometric mean expression of the housekeeper genes

Figure Supplementary 2. Bulk RNA-seq results of the TNF pathway

Figure Supplementary 3. mRNA expression of the TNFSF receptors by diagnosis

Figure Supplementary 4. Ramified-like FAS+ cells in the dorsal midbrain

References Supplementary

Methods Supplementary

Human Postmortem Tissue Collection and Midbrain Cohort Demographics

Human post-mortem midbrain tissue was obtained from the New South Wales Brain Tissue Resource Centre (TRC) and Stanley Medical Research Institute (SMRI) Array cohorts. The whole mRNA cohort included 61 healthy controls, 63 schizophrenia cases, and 33 bipolar disorder cases. Age, postmortem interval (PMI), and RNA integrity number (RIN) did not significantly differ between diagnostic groups. Compared to the control group, the bipolar disorder and schizophrenia groups had lower brain pH (both $p \le 0.001$). In addition, the bipolar disorder group had fewer males compared to the control and schizophrenia groups and fewer cases that died by suicide compared to the schizophrenia group (all $p \le 0.05$), potentially due to a smaller sample size. Consistent with clinical observations, the bipolar disorder group had a lower average lifetime chlorpromazine equivalent dose ($p=0.001$) and had fewer years with illness ($p=0.05$) compared to the schizophrenia group. Our study was approved by the Human Research Ethics Committee at the University of NSW (#HREC: HC230253). All cases in the cohorts were previously categorized into low or high-inflammation subgroups using a two-step recursive clustering analysis based on mRNA expression of *SERPINA3*, Interleukin *(IL)-1β*, *IL-6*, and *TNF*(1, 2). The combined cohort consisted of 59 low-inflammation control (CTRL LI), 2 high-inflammation control (CTRL HI), 34 low-inflammation schizophrenia (SZ HI), 29 high-inflammation schizophrenia (SZ HI), 24 low-inflammation bipolar disorder (BPD LI), and 9 high-inflammation bipolar disorder (BPD HI) cases. 2 high-inflammation control cases were not included in inflammation subgroup statistical analyses due to small sample size. Cases with any underpinning inflammation-related condition were excluded from the study. Cases for the RNA-seq and immunohistochemistry experiments were taken from the whole mRNA cohort.

RNA extraction

Total RNA was extracted from fresh frozen ventral midbrain tissue using the TRIzol (Invitrogen, Carlsbad, CA, United States, 15596026) extraction method as previously described(3). RNA concentration (on nanodrop) and quality were determined by Agilent Technologies 2100 Bioanalyzer.

Midbrain bulk RNA sequencing, differential expression and pathway enrichment analyses

The bulk RNA sequencing analysis included 20 control and 40 schizophrenia cases from the whole mRNA cohort. We conducted paired-end sequencing of 100 bp lengths using the NovaSeq 6000 System, with all sample libraries run as replicates across multiple lanes to minimise batch effects and achieve higher sequence depth. An average of 296M total reads and 153M unique paired-end reads were attained per sample, with the average Phred quality score over 34 across all samples and positions before and after trimming. Illumina-specific adapters and low-quality reads were trimmed with Trimmomatic. An average of 92.6% of reads were aligned to Genome Reference Consortium Human Build 38 (GRCh38) using STAR aligner, followed by assigning mapped reads to genomic features using FeatureCounts. The gene counts were analyzed for differential expression (DE) across groups after filtering low-expressed genes using DESeq2 (adjusted $p<0.05$ using Benjamini-Hochberg adjustment).

DE analysis was performed using the DESeq2 package in R. Raw count data and corresponding sample were loaded into a DESeq data set object using the formula "~Diagnosis" as the design. Genes with fewer than 10 reads in less than 2 samples were filtered out to reduce low-expression noise. Normalized counts were extracted using the "counts()" function with normalization enabled. These counts were then annotated using gene names corresponding to Ensembl code. DESeq2 results were obtained by comparing schizophrenia to control groups. Significant genes were filtered using an adjusted p-value cutoff of 0.05 and annotated with gene information. Gene identifiers were converted to Entrez IDs using the org.Hs.eg.db database to enable pathway enrichment analysis. KEGG pathway enrichment was performed using clusterProfiler::enrichKEGG function. Gene expression fold changes were also calculated and matched by Entrez ID. To improve the readability of pathway results and enable further protein ID mapping, multiple identifier conversions were conducted. The bitr_kegg and bitr() functions were used for the conversions. All packages used include: DESeq2, org.Hs.eg.db, clusterProfiler, gage, gageData, pathview. KEGG enrichment sets were loaded from gageData and restricted to signaling/metabolic pathways using the sigmet.idx.hs index.This pipeline supports biological interpretation of differentially expressed genes between diagnoses by identifying significantly enriched pathways based on fold-change profiles and Entrez IDs.

Complementary DNA synthesis, and quantitative PCR

Complementary DNA (cDNA) synthesis was performed from 1 µg total RNA per case using SuperScript III First-Strand Synthesis kit (Life Technologies, Scoresby, VIC, Australia). The mRNA expression of the TNFSF markers was measured by reverse transcriptase-quantitative PCR using the Fluidigm BioMarkTM HD system (South San Francisco, CA, United States) at the Ramaciotti Centre for Genomics (Kensington, NSW, Australia) using pre-designed Taqman Gene Expression Assays. mRNA expression of each gene was calculated by normalizing the concentration (as determined by the relative standard curve method) of the target transcript to the geometric mean of three housekeeper transcripts, glyceraldehyde 3-phosphate dehydrogenase (*GAPDH*) (Hs99999905_m1), Beta-glucuronidase (*GUSB*) (Hs99999908_m1), and ubiquitin C (*UBC*) (Hs00824723_m1). There were no statistically significant changes in the geometric mean of the housekeeper genes between diagnostic groups (H(2, 157)=4.94, p=0.084).

Midbrain snRNA-seq analyses

Human postmortem samples, nuclei isolation, and snRNA sequencing steps were detailed previously(4). The snRNA-seq included 14 control and 20 schizophrenia cases. Single-nucleus cDNA libraries were constructed using the 10X Genomics Chromium single-cell 3' reagents kit v3 according to the user guide. All the sequencing reads were processed using 10X Genomics Cell Ranger 7.0.1 by aligning the reads to GRCh38 human genome release 110 (downloaded from Ensembl, https://ftp.ensembl.org/pub/) along with basic quality control analyses. Both exonic and intronic reads were included in the analysis by Cell Ranger. The mean sequencing depth was 223 million reads per sample, and the mean reads per nucleus was 43,263. The mean estimated number of nuclei is 5,184 per sample, and the mean total detected genes was 38,326. The Scrublet package was used to identify and remove doublets from the raw counts with default

settings. The count matrix generated from 10X Genomics with doublet information was imported into R with the Seurat v5 package, whereas the CellRanger's filtered feature_bc_matrix output was used to analyze the data. The nuclei that contain >5% mitochondrial RNA were removed. The counts from all the cases were log-normalized using Seurat, followed by data integration using fast reciprocal PCA (rPCA). The integrated Seurat object was used to run unsupervised clustering analyses using FindNeighbors (dims=1:30, k.param=10) and FindClusters (resolution=0.4) functions to identify cell types/clusters based on marker genes. FAS, TNFR1, and TNFR2 gene expression levels were visualized using Uniform Manifold Approximation and Projection (UMAP) plots in Seurat. The expression of TNFSF receptors *DR4* and *TWEAKR* was deemed too low to be accurately mapped.

FAS immunostaining

Fresh frozen midbrain horizontal sections (14µm) were used for brightfield immunohistochemistry to detect FAS+ cells. Negative control slides incubated in the absence of primary antibodies were included in all experiments. One midbrain section per case (Control n=9, schizophrenia n=18 and bipolar disorder n=18, Supplementary Table 4) was fixed in 4% paraformaldehyde and treated with 3:1 methanol 30% and hydrogen peroxide for 20 min to inhibit endogenous peroxidase activity. Slides were blocked in 10% normal goat serum (S-1000, Vector Laboratories, Burlingame, CA) and incubated (4°C overnight) with primary antibody (mouse anti-FAS 1:250, sc-8009, Santa Cruz). Tissue was washed with phosphate-buffered saline and incubated in secondary antibody (goat anti-mouse IgG biotinylated, 1:500, BA-9200, Vector Laboratories) before incubation with avidin-biotin-peroxidase complex (VectaStain, PK-4000, Vector Laboratories) and visualization with a 3,3-diaminobenzidine (DAB) reaction. Slides were washed, dehydrated, counterstained with 0.25% Thionin, cleared in xylene, and coverslipped with Permount Mounting Medium (KIRWAN QLD 4817, Australia). FAS+ cells were defined as positive when DAB staining colocalized with nuclear thionin staining and both were found in the same focal plane.

Statistical analysis

All experiments were performed blind to case designation. The Shapiro-Wilk test was used to evaluate the normality of our data. For analyses between groups, ANOVA/ANCOVA + LSD post hoc tests were used on the normally distributed continuous variables, whereas the Kruskal–Wallis+ Dunn-Bonferroni post-hoc test, or Quade's ANCOVA, were used on the non-normally distributed continuous variables. Categorical variables were tested with the Pearson's chi-squared (X2) test. Correlation analyses were performed using the parametric Pearson (r) and the non-parametric Spearman's rank (rho) correlation coefficient tests for normally and non-normally distributed variables, respectively. Those demographic variables that had significant correlations with dependent variables were used as covariates in between-group analyses. Brain pH was not included as a covariate since pH is lower in postmortem samples of schizophrenia brains(5) and acidosis is typical of inflamed tissue(6). Statistical analyses were performed using SPSS Statistics (version 25; IBM, Armonk, NY, USA). Based on an exclusion criterion of mean values per group, two standard deviation outlier cases were removed from the analysis. mRNA data were square root transformed when needed. Data graphs were plotted using the mean ± its standard error (SEM) using the GraphPad Prism (8.0.1, San Diego, California, USA) software.

Tables Supplementary

Table Supplementary 1. Demographics of the bulk RNA-seq cohort

**Demographics**	**Control (n = 20)**	**Schizophrenia (n = 40)**	**Statistics**
Age in years ± s.d. (range)	44.30 ± 8.91 (22-60)	45.05 ± 10.26 (19-67)	$T_{(58)}$=-.278, p=0.782
Sex	5F:15M	11F:29M	$\chi^2_{(1)}$ = 0.43, p=0.836
Brain pH ± s.d.	6.68 ± 0.25	6.51 ± 0.22	**$T_{(58)}$=2.64, p=0.010**
PMI (hours) ± s.d.	31.25 ± 12.66	33.82 ± 16.86	$T_{(58)}$=-.602, p=0.549
RIN ± s.d.	6.47 ± 1.05	6.29 ± 1.53	$U_{(60)}$=398, p=0.975
Age (years) at onset ± s.d.	—	22.50 ± 6.89	—
Duration of illness (years) ± s.d.	—	22.90 ± 11.37	—
Lifetime chlorpromazine equivalent dose (mg)	—	4860447.36 ± 6007828.46	—

PMI, post-mortem interval; *RIN*, RNA integrity number. Bold, statistical significance.

Table Supplementary 2. Taqman Gene Expression Assays used

Gene	Gene Name	Assay ID
BAX	BCL2 Associated X, Apoptosis Regulator/BCL2L4	Hs00180269_m1
BCL2	BCL2, Apoptosis Regulator	Hs00608023_m1
BID	Bh3 Interacting Domain Death Agonist	Hs00609632_m1
CASP1	Caspase 1	Hs00354836_m1
CASP3	Caspase 3	Hs00234387_m1
CASP7	Caspase 7	Hs00169152_m1
CASP8	Caspase 8	Hs01018151_m1
CASP9	Caspase 9	Hs00962278_m1
DR4/TNFRSF10A	Death Receptor 4/ TNF Receptor Superfamily Member 10A	Hs00269492_m1
FAS/TNFRSF6	Fas Cell Surface Death Receptor/ TNF Receptor Superfamily Member 6	Hs00236330_m1
GFAP	Glial Fibrillary Acidic Protein	Hs00909236_m1
MCL1	BCL2 Family Apoptosis Regulator	Hs01050896_m1
P53	Microtubule-Associated Protein Light Chain I / Tumor Protein P53	Hs01034249_m1
TNFR1/TNFRSF1A	TNF Receptor Superfamily Member 1A	Hs01042313_m1
TNFR2/TNFRSF1B	TNF Receptor Superfamily Member 1B	Hs00961750_m1
TWEAKR/TNFRSF12A	TNF Receptor Superfamily Member 12A	Hs00171993_m1

Table Supplementary 3. Demographics of the snRNA-seq cohort

Demographics	Control (n = 14)	Schizophrenia (n = 20)	Statistics
Age in years ± s.d. (range)	44.71 ± 7.84 (34-60)	41.35 ± 9.56 (19-54)	$T_{(32)}$=1.08, p=0.286
Sex	4F:10M	3F:17M	$\chi^2_{(1)}$ = 0.92, p=0.335
Brain pH ± s.d.	6.60 ± 0.30	6.43 ± 0.21	$T_{(32)}$=1.91, p=0.064
PMI (hours) ± s.d.	30.42 ± 12.23	30.90 ± 16.81	$U_{(34)}$=137, p=0.916
RIN ± s.d.	6.02 ± 1.07	6.48 ± 1.25	$T_{(32)}$=-1.10, p=0.279
Brain weight (g) ± s.d.	1425.35 ± 155.21	1451.75 ± 105.89	$T_{(32)}$=-0.591, p=0.559
Age (years) at onset ± s.d.	—	21.50 ± 5.41	—
Duration of illness (years) ± s.d.	—	19.80 ± 8.00	—
Lifetime chlorpromazine equivalent dose (mg)	—	88925 ± 81419.36	—

PMI, post-mortem interval; *RIN*, RNA integrity number. Bold, statistical significance.

Table Supplementary 4. Demographics of the Immunohistochemistry cohort

Demographics	Control (n = 9)	Schizophrenia (n = 18)	Bipolar Disorder (n = 18)	Statistics
Age in years ± s.d. (range)	43.77 ± 6.47 (32-53)	44.72 ± 8.02 (31-59)	44.38 ± 10.44 (19–64)	$F_{(2, 42)}$=0.03, p=0.966
Sex	2F:7M	5F:13M	12F:6M	**$\chi^2_{(2)}$= 7.42, *p*=0.024**
Brain pH ± s.d.	6.50 ± 0.36	6.45 ± 0.27	6.30 ± 0.29	$F_{(2, 157)}$=1.69, *p*=0.196
PMI (hours) ± s.d.	30.44 ± 10.71	32.16 ± 11.75	33.77 ± 16.68	$F_{(2, 42)}$=0.18, *p*=0.834
RIN ± s.d.	5.96 ± 1.06	6.19 ± 1.00	6.02 ± 1.30	$F_{(2, 42)}$=0.15, *p*=0.856
Brain weight (g) ± s.d.	1404.88 ± 151.22	1444.11 ± 123.59	1348.83 ± 144.85	$F_{(2, 42)}$=2.16, *p*=0.128
Age (years) at onset ± s.d.$	—	19.44 ± 3.85	24 ± 7.72	$U_{(35)}$= 102, *p*=0.091
Duration of illness (years) ± s.d.$	—	25.22 ± 8.11	20.27 ± 11.13	$T_{(34)}$= 1.52, *p*=0.137
Lifetime chlorpromazine equivalent dose (mg)$	—	112500 ± 111780.37	15816.66 ± 30119.06	**$U_{(36)}$= 27.50, *p*<0.001**

PMI, post-mortem interval; *RIN*, RNA integrity number. *$*, statistical analysis between psychiatric cases only. Bold, statistical significance.

Table Supplementary 5. Correlations between demographic and dependent variables in the whole mRNA cohort

Demographics	DR4/TNFRSF10A	FAS/ TNFRSF6	TWEAKR/ TNFRSF12A	TNFR1/ TNFRSF1A	TNFR2/ TNFRSF1B	GFAP
Age in years	*rho*=0.039, *p*=0.633	*rho*=0.083, *p*=0.317	*rho*=0.063, *p*=0.450	*rho*=-0.034, *p*=0.682	*rho*=0.115, *p*=0.163	*rho*=-0.015, *p*=0.857
Brain pH	***rho*=-0.326, *p*=4.74E-5**	***rho*=-0.242, *p*=0.003**	***rho*=-0.209, *p*=0.012**	***rho*=-0.273, *p*=0.001**	***rho*=-0.284, *p*=4.39E-4**	***rho*=-0.306, *p*<.001**
PMI (hours)	*rho*=-0.109, *p*=0.182	***rho*=-.168, *p*=0.042**	*rho*=0.084, *p*=0.312	*rho*=0.074, *p*=0.372	*rho*=-0.007, *p*=0.936	*rho*=-0.032, *p*=0.703
RIN	*rho*=-0.146, *p*=0.075	*rho*=-0.153, *p*=0.065	*rho*=-0.029, *p*=0.729	***rho*=-0.217, *p*=0.008**	*rho*=-0.095, *p*=0.249	***rho*=-0.176, *p*=0.035**
Brain Weight (mg)	*rho*=-0.071, *p*=0.463	*rho*=0.025, *p*=0.796	*rho*=-0.126, *p*=0.199	*rho*=0.028, *p*=0.778	*rho*=-0.068, *p*=0.483	*rho*=-0.096, *p*=0.339

PMI, post-mortem interval; *RIN*, RNA integrity number. Bold, statistical significance.

Table Supplementary 6. Correlations between demographic and dependent variables in the SMRI mRNA cohort

Demographics	*BAX*	*BCL2*	*BID*	*CASP3*	*CASP9*	*CASP1*	*CASP7*	*CASP8*	*MCL1*	*P53*
Age in years	*r*=0.041, *p*=0.696	*rho*=-0.021, *p*=0.839	*r*=0.007, *p*=0.945	***r*=-0.230, *p*=0.024**	*rho*=0.015, *p*=0.887	*r*=-0.092, *p*=0.368	*rho*=0.179, *p*=0.084	*r*=0.103, *p*=0.321	*rho*=-0.075, *p*=0.465	*r*=-0.111, *p*=0.284
Brain pH	***rho*=-0.276, *p*=0.007**	***rho*=-0.526, *p*=$3.69E^{-8}$**	*rho*=0.070, *p*=0.496	*rho*=-0.085, *p*=0.409	***rho*=-0.331, *p*=0.001**	*rho*=0.060, *p*=0.562	***rho*=-0.253, *p*=0.014**	*rho*=0.162, *p*=0.116	***rho*=-0.497, *p*=$2.30E^{-7}$**	***rho*=-0.273, *p*=0.007**
PMI (hours)	*rho*=0.021, *p*=0.843	*rho*=-0.156, *p*=0.130	*rho*=0.061, *p*=0.548	***rho*=-0.227, *p*=0.026**	***rho*=-0.027, *p*=0.025**	*rho*=-0.090, *p*=0.379	*rho*=-0.013, *p*=0.905	*rho*=-0.104, *p*=0.315	*rho*=-0.140, *p*=0.171	***rho*=-0.284, *p*=0.005**
RIN	*r*=0.030, *p*=0.776	***rho*=-0.213, *p*=0.038**	*r*=-0.051, *p*=0.619	***r*=0.240, *p*=0.018**	*rho*=0.038, *p*=0.713	*r*=0.031, *p*=0.766	***rho*=-0.360, *p*<0.001**	*r*=0.088, *p*=0.398	*rho*=-0.057, *p*=0.576	*r*=0.022, *p*=0.835
Brain Weight (mg)	*r*=-0.015, *p*=0.888	*rho*=-0.154, *p*=0.135	*r*=-0.036, *p*=0.724	*r*=0.071, *p*=0.489	*rho*=-0.140, *p*=0.171	*r*=0.034, *p*=0.744	*rho*=-0.158, *p*=0.129	*r*=0.054, *p*=0.605	*rho*=-0.182, *p*=0.074	*r*=-0.158, *p*=0.125
Lifetime Alcohol Use	***rho*=0.230, *p*=0.026**	*rho*=0.020, *p*=0.848	*rho*=0.095, *p*=0.354	*rho*=0.017, *p*=0.871	*rho*=0.020, *p*=0.844	***rho*=-0.223, *p*=0.029**	*rho*=-0.129, *p*=0.219	***rho*=-0.208, *p*=0.044**	*rho*=0.173, *p*=0.092	*rho*=-0.015, *p*=0.889
Lifetime Drug Use	***rho*=0.255, *p*=0.014**	*rho*=0.054, *p*=0.601	*rho*=0.145, *p*=0.158	*rho*=0.128, *p*=0.220	*rho*=0.119, *p*=0.250	*rho*=-0.107, *p*=0.300	*rho*=-0.144, *p*=0.171	*rho*=-0.121, *p*=0.249	***rho*=0.226, *p*=0.027**	*rho*=0.134, *p*=0.200
Smoking at Time of Death	*rho*=0.051, *p*=0.684	*rho*=0.130, *p*=0.297	*rho*=0.050, *p*=0.697	*rho*=-0.074, *p*=0.559	*rho*=0.192, *p*=0.122	*rho*=-0.048, *p*=0.705	*rho*=0.047, *p*=0.715	*rho*=0.159, *p*=0.217	*rho*=0.210, *p*=0.093	*rho*=0.192, *p*=0.132

PMI, post-mortem interval; *RIN*, RNA integrity number. Bold, statistical significance.

Table Supplementary 7. Comparisons of our dependent variables when including brain pH as a covariate

mRNA	Covariates	Statistical test result	Post hoc
TNFR1	RIN and brain pH	**Quade's ANCOVA $F_{(4, 143)}$=4.11, *p*=0.003**	***LI CTRL vs HI SZ: *p*= 0.001**<br>***LI CTRL vs LI BPD: *p*=0.006**
TNFR2	Brain pH	**Quade's ANCOVA $F_{(4, 144)}$=11.71, *p*=2.88E-8**	***LI CTRL vs HI SZ: *p*= 4.08E-9**<br>***LI SZ vs HI SZ: *p*= 2.68E-7**<br>***HI SZ vs LI BPD: *p*=0.00006**
FAS	PMI and brain pH	**Quade's ANCOVA $F_{(4, 142)}$=8.75, *p*=2.0E-4**	***LI CTRL vs HI SZ: *p*= 2.85E-7**<br>***LI SZ vs HI SZ: *p*= 0.000012**<br>***HI SZ vs LI BPD: *p*=0.000011**
DR4	Brain pH	**Quade's ANCOVA $F_{(4, 145)}$=9.81, *p*=4.67E-7**	***LI CTRL vs HI SZ: *p*= 6.61E-8**<br>***LI SZ vs HI SZ: *p*= 0.000005**<br>***HI SZ vs LI BPD: *p*=0.000006**
TWEAKR	Brain pH	**Quade's ANCOVA $F_{(4, 140)}$=5.04, *p*=0.001**	***LI CTRL vs HI SZ: *p*= 0.000073**<br>***LI CTRL vs HI BPD: *p*= 0.016**<br>***LI SZ vs HI SZ: *p*= 0.012**<br>***HI SZ vs LI BPD: *p*=0.005**
GFAP	RIN and brain pH	**Quade's ANCOVA $F_{(4, 138)}$=3.69, *p*=0.007**	***LI CTRL vs HI SZ: *p*= 0.004**<br>***LI SZ vs HI SZ: *p*= 0.001**<br>***HI SZ vs LI BPD: *p*=0.006**
BAX	Brain pH	ANCOVA $F_{(4, 88)}$=1.89, *p*=0.119	
BID	Brain pH	ANCOVA $F_{(4, 90)}$=1.44, *p*=0.226	
BCL2	RIN and brain pH	ANCOVA $F_{(4, 87)}$=1.63, *p*=0.172	
CASP1	Brain pH	**ANCOVA $F_{(4, 88)}$=3.59, *p*=0.009**	***LI SZ vs HI SZ: *p*= 0.009**<br>***HI SZ vs LI BPD; *p*<0.001**
CASP3	Brain pH	ANCOVA $F_{(4, 87)}$=2.71, *p*=0.035	p-values >.016 (adjusted)
CASP7	RIN and brain pH	**Quade's ANCOVA $F_{(4, 89)}$=3.71, *p*=0.008**	***LI CTRL vs HI SZ: *p*= 0.003**<br>***LI SZ vs HI SZ: *p*= 0.006**<br>***HI SZ vs LI BPD: *p*=0.002**<br>***HI SZ vs HI BPD: *p*=0.002**
CASP8	Brain pH	**ANCOVA $F_{(4, 86)}$=3.85, *p*=0.006**	***LI CTRL vs HI SZ: *p*= 0.007**<br>***LI SZ vs HI SZ: *p*= 0.002**<br>***HI SZ vs LI BPD: *p*<0.001**<br>***HI SZ vs HI BPD: *p*=0.010**
CASP9	PMI and brain pH	**ANCOVA $F_{(4, 88)}$=2.82, *p*=0.030**	***HI SZ vs LI BPD: *p*=0.002**
MCL-1	Brain pH	**Quade's ANCOVA $F_{(4, 90)}$=3.32, *p*=0.014**	***LI CTRL vs HI SZ: *p*= 0.007**<br>***LI SZ vs HI SZ: *p*= 0.003**<br>***HI SZ vs LI BPD: *p*=0.011**
P53	Brain pH	**Quade's ANCOVA $F_{(4, 88)}$=3.20, *p*=0.017**	***HI SZ vs LI BPD: *p*=0.002**<br>***HI SZ vs HI BPD: *p*=0.012**

PMI, post-mortem interval; RIN, RNA integrity number. Bold, statistical significance.

Table Supplementary 8. Comparisons of our dependent variables by sex

mRNA	*t*-test result
TNFR1	$t_{(146)}$=.430, *p*=.669
TNFR2	$t_{(147)}$=-.245, *p*=.807
FAS	$t_{(145)}$=.674, *p*=.502
DR4	$t_{(143)}$=1.16, *p*=.246
TWEAKR	$t_{(148)}$=.824, *p*=.411
GFAP	$t_{(141)}$=.276, *p*=.783
BAX	$t_{(94)}$=-.114, *p*=.910
BID	$t_{(94)}$=-1.14, *p*=.256
BCL2	$t_{(92)}$=.876, *p*=.383
CASP1	$t_{(95)}$=-1.47, *p*=.144
CASP3	$t_{(92)}$=-1.17, *p*=.241
CASP7	$t_{(92)}$=.595, *p*=.553
CASP8	$t_{(93)}$=-.554, *p*=.581
CASP9	$t_{(93)}$=.012, *p*=.991
MCL-1	$t_{(93)}$=.373, *p*=.710
P53	$t_{(91)}$=.996, *p*=.332

Table Supplementary 9. Differentially expressed genes between schizophrenia and control by bulk RNA-seq

Gene name	Log2FoldChange	*p*-value	*p*-adj
TTR	-3.792533646	1.81E-13	7.41E-09
SLC5A5	-2.886493379	7.99E-12	1.64E-07
LRG1	2.475973919	8.78E-10	1.20E-05
EDN1	**1.728333287**	**2.11E-09**	**1.87E-05**
HDC	-4.147012504	2.28E-09	1.87E-05
CHI3L2	1.918930285	1.83E-08	0.000125
SIX6	-2.729353992	2.54E-08	0.000148
ISYNA1	-0.755046907	4.61E-08	0.00022
SPAG6	-1.381971514	4.84E-08	0.00022
SERPINA3	2.874019822	5.45E-08	0.000221
CD163	1.911642164	5.94E-08	0.000221
AC061992.1	3.223180936	8.17E-08	0.000279
VSIG4	1.493189791	1.12E-07	0.000352
SLAMF8	2.424659945	1.30E-07	0.00038
WIF1	-1.421569578	1.40E-07	0.000382
CHI3L1	2.087561074	1.70E-07	0.000436
APOL4	1.995329455	2.32E-07	0.000559
DLX2	-2.913582026	2.63E-07	0.000599
AL929091.1	1.805321605	3.88E-07	0.000836
MFRP	-1.796600894	4.28E-07	0.000877
SOCS3	**2.246415192**	**4.81E-07**	**0.000935**
AL049839.2	1.27011752	5.02E-07	0.000935
TMEM72	-1.107314891	5.44E-07	0.000969
EPHA4	-0.673753656	8.46E-07	0.001444
HAMP	2.103878822	9.21E-07	0.001508
SPAAR	0.907959216	1.03E-06	0.001622
BCL2A1	1.924372053	1.35E-06	0.001948

HILPDA	1.71267454	1.35E-06	0.001948
OSMR-AS1	1.220049609	1.38E-06	0.001948
GRHL3	1.879075509	1.45E-06	0.001976
S100A9	2.017790081	1.51E-06	0.001994
SLCO4A1	1.490510301	1.72E-06	0.002166
C14orf180	-1.354472444	1.75E-06	0.002166
KRT5	-2.303825383	1.84E-06	0.002212
IL1R2	2.33644714	2.12E-06	0.002483
AL390755.1	1.448347728	2.20E-06	0.002508
IFITM3	0.738019034	2.48E-06	0.00275
MC3R	-2.678439386	2.73E-06	0.002936
OSMR	1.210133749	2.80E-06	0.002936
SERPINA5	1.115218455	3.11E-06	0.003184
DLX5	-1.27703073	3.24E-06	0.003223
FOLR1	-1.577674723	3.31E-06	0.003223
MPZL2	1.330410352	3.55E-06	0.00331
PRLR	-1.321382925	3.56E-06	0.00331
ABCA4	-1.228495507	4.00E-06	0.003637
PLA2G2A	2.018276631	4.22E-06	0.00376
AL449403.2	0.927372777	4.36E-06	0.003796
LINC02119	1.526556194	4.51E-06	0.003846
IFITM2	0.911909668	4.75E-06	0.003966
SECTM1	1.42377088	5.10E-06	0.004159
SFN	1.975318514	5.19E-06	0.004159
CSF3	2.827601862	5.34E-06	0.004159
POMC	-1.198744047	5.45E-06	0.004159
SLC11A1	1.546675531	5.53E-06	0.004159
C1RL	0.621789766	5.59E-06	0.004159
REELD1	0.892093049	6.42E-06	0.004665
KIF19	1.058719263	6.51E-06	0.004665

AL355974.2	1.544387454	6.70E-06	0.004665
AL449403.1	0.821314969	6.78E-06	0.004665
LINC01010	1.161963128	6.84E-06	0.004665
MTHFD2	0.753552153	8.39E-06	0.005554
ACSL5	0.788270588	8.41E-06	0.005554
JAK3	0.821736359	8.73E-06	0.005674
S100A8	2.106514345	9.45E-06	0.006045
S100A3	1.497050229	9.59E-06	0.006045
KIAA0040	1.436199918	9.90E-06	0.006123
TCAF2	1.127251736	1.00E-05	0.006123
TIMP1	0.883251575	1.06E-05	0.006363
AC063919.1	0.900771441	1.10E-05	0.006508
OCA2	-0.898880937	1.14E-05	0.006664
MT1A	2.015078456	1.24E-05	0.007057
AC241377.2	1.275198101	1.25E-05	0.007057
TAC3	-1.2487949	1.26E-05	0.007057
ANKRD22	1.866635033	1.28E-05	0.007057
AC007906.2	-1.522564063	1.32E-05	0.007078
AL390755.2	1.600631777	1.33E-05	0.007078
AC091435.2	1.250960711	1.33E-05	0.007078
LILRA5	1.810930762	1.36E-05	0.007158
SLC1A5	0.866325916	1.39E-05	0.007158
AL355974.1	1.687765438	1.40E-05	0.007158
PRKX	0.533306324	1.44E-05	0.007258
MAPK15	-1.7540324	1.47E-05	0.00735
C1R	0.764944142	1.59E-05	0.007669
KCNF1	-0.796384457	1.59E-05	0.007669
SLC39A14	1.32337325	1.60E-05	0.007669
CCN4	0.658073653	1.61E-05	0.007669
SKOR2	-2.668097042	1.70E-05	0.007975

PI15	1.446432223	1.71E-05	0.007975
FKBP5	1.181173424	1.90E-05	0.008705
TEAD4	0.985946583	1.92E-05	0.008705
MT1X	1.229759181	1.93E-05	0.008705
GMNC	-0.998809021	1.96E-05	0.008713
DLX1	-1.64334753	2.03E-05	0.008925
SULT1B1	0.994997757	2.32E-05	0.010102
CCL2	**1.649802305**	**2.37E-05**	**0.010233**
AC010478.1	-2.380491913	2.44E-05	0.010404
PRSS23	0.485699008	2.61E-05	0.01102
SLC22A8	-1.417247675	2.67E-05	0.011139
ADTRP	-0.994961623	2.80E-05	0.011586
HPD	-1.108856127	2.91E-05	0.0119
IL6	**1.692788149**	**2.98E-05**	**0.012091**
CYP51A1P2	0.756949497	3.19E-05	0.012766
CRHR2	-0.807944448	3.21E-05	0.012766
LIF	**2.075120618**	**3.25E-05**	**0.012776**
IL1RL1	2.749202986	3.28E-05	0.012776
AC010368.1	-2.274981203	3.51E-05	0.013549
AL355812.1	-1.281737378	3.59E-05	0.013719
AFDN	0.170907094	3.62E-05	0.013719
EMP1	1.403373246	3.92E-05	0.014614
LILRA6	1.200133674	3.93E-05	0.014614
RNASE2	1.139496887	4.05E-05	0.014945
CD177	1.908678579	4.09E-05	0.014946
RN7SKP296	0.800511669	4.18E-05	0.015065
DNAAF1	-0.634280872	4.19E-05	0.015065
GPR84	1.172768629	4.34E-05	0.015472
AC105910.1	1.49608718	4.45E-05	0.015651
KCNMB4	0.677030636	4.47E-05	0.015651

C14orf39	-0.883646788	4.52E-05	0.015699
PIM1	0.778152343	4.60E-05	0.015821
ADGB	-1.281880168	4.76E-05	0.016097
LOXL2	0.786371563	4.79E-05	0.016097
AC099548.2	0.99621016	4.80E-05	0.016097
LRRC18	-0.894172037	4.86E-05	0.016097
MIR4527HG	1.274703034	4.87E-05	0.016097
ACKR1	1.053070526	5.19E-05	0.01699
TGM2	1.030266203	5.38E-05	0.017494
MT1JP	1.676267101	5.44E-05	0.017556
PTK7	-0.404849846	5.63E-05	0.017917
AL359762.1	0.820665268	5.64E-05	0.017917
KCNJ15	1.095543992	5.88E-05	0.018518
LINC02159	-1.965296864	6.04E-05	0.018894
SELE	**1.493988147**	**6.33E-05**	**0.019626**
DEPP1	1.078805335	6.43E-05	0.019808
PTGES3P1	0.956159017	6.48E-05	0.01981
CCDC114	-0.751285712	6.65E-05	0.02017
SAMHD1	0.413127437	6.88E-05	0.020727
FCGR1A	1.16115979	7.10E-05	0.021137
MYO1G	1.220715519	7.17E-05	0.021137
WFIKKN2	-1.013558241	7.17E-05	0.021137
FFAR2	1.658408417	7.31E-05	0.021395
CSN1S1	-1.624654354	7.37E-05	0.021412
AL355103.1	-1.797768904	7.70E-05	0.021839
PITX2	-1.765051292	7.73E-05	0.021839
AC125807.2	0.95884443	7.76E-05	0.021839
MIR1911	-1.883252742	7.76E-05	0.021839
AC132216.1	1.583865276	7.79E-05	0.021839
TNFRSF1A	**0.594552896**	**7.87E-05**	**0.021938**

AC103563.9	0.567852088	7.98E-05	0.022068
DLX6	-1.403596842	8.08E-05	0.022136
MT1M	1.093522944	8.11E-05	0.022136
TCIM	0.924483668	8.34E-05	0.022612
RF02250	-2.061946212	8.51E-05	0.022705
LINC02660	-2.899324827	8.53E-05	0.022705
AC007877.1	1.507765222	8.54E-05	0.022705
CPNE9	-1.118812014	8.59E-05	0.022705
AC019257.1	0.533986777	9.10E-05	0.023836
AC103858.1	-0.790805951	9.15E-05	0.023836
FZD8	-0.628397082	9.20E-05	0.023836
AL445426.1	0.767310414	9.30E-05	0.023966
AC188617.1	1.217084326	9.45E-05	0.024104
IL4R	0.962023625	9.48E-05	0.024104
OXTR	1.041864883	9.89E-05	0.025012
C1S	0.497146971	0.000101	0.02533
AC104809.2	-2.124206159	0.000102	0.02542
TTLL10	-1.864732963	0.000103	0.025589
TRPC3	-0.412724016	0.000104	0.025596
ISL1	-2.482424726	0.000104	0.025596
AC091180.2	0.553602412	0.000105	0.025646
EGF	0.394406266	0.000106	0.025646
RNU6-894P	1.375022059	0.000107	0.02589
CD44-AS1	1.255271756	0.000108	0.025961
STEAP4	0.927927869	0.00011	0.026124
IDH2-DT	0.586590576	0.000111	0.026193
SYT6	-0.671706524	0.000112	0.026451
C1RL-AS1	0.627693969	0.000115	0.027029
AP004290.1	0.380424446	0.000119	0.027719
RNF144B	0.34746188	0.000121	0.027999

FPR1	1.250073269	0.000134	0.030727
DNAI1	-0.524751482	0.000145	0.032854
RASEF	-0.800170019	0.000145	0.032854
AL358216.1	0.446928836	0.000145	0.032854
MAP3K6	0.560487709	0.000147	0.033029
F3	1.106728162	0.000148	0.033029
ANGPTL4	1.163755443	0.000149	0.033066
SMIM25	1.149129473	0.000153	0.033819
SPOCD1	1.130012598	0.000154	0.033819
ITPKC	0.857609353	0.000156	0.034112
KBTBD11	0.271542017	0.000157	0.034118
PECAM1	0.561148986	0.000157	0.034118
CCIN	1.153825047	0.000159	0.034174
AL161757.4	-0.871212299	0.000161	0.034439
COL9A1	-0.538881521	0.000163	0.034754
MS4A8	-1.442636382	0.000167	0.035466
CFAP73	-0.874859767	0.00017	0.035825
AC003989.2	0.442254316	0.000172	0.035831
AC004264.1	2.581414566	0.000172	0.035831
BCL3	**0.778039424**	**0.000172**	**0.035831**
AC112236.3	0.74483426	0.000173	0.035839
AC078955.1	0.760957122	0.000174	0.035839
FPR2	1.442033603	0.000178	0.036452
CD44	0.671251798	0.000181	0.036929
CYP4Z2P	1.02890521	0.000185	0.037405
MELTF	-0.593202049	0.000186	0.037405
AP000770.1	0.890007395	0.000187	0.037405
SOD2	0.692143905	0.000188	0.037405
SLCO4A1-AS1	1.130817846	0.00019	0.037405
CASP12	-0.814149258	0.00019	0.037405

MAFB	0.73147542	0.00019	0.037405
ADAMTS9	1.203913914	0.000194	0.037792
AL157373.2	-1.604771111	0.000194	0.037792
CARTPT	-1.93092538	0.000197	0.03833
BMP2	0.610336594	0.000206	0.039794
AC015819.1	0.712519705	0.000208	0.039988
APOL6	0.642230447	0.000209	0.040059
KLF10	0.648655064	0.000212	0.040414
MRGPRE	-0.813848704	0.000217	0.041099
C2CD4A	1.485747322	0.000218	0.041235
SMTNL1	0.762760509	0.000223	0.04192
SBNO2	0.669303363	0.000226	0.042214
HRK	-0.424559241	0.000232	0.043248
RNF24	0.260110581	0.000234	0.043441
AL512329.2	0.645506299	0.000239	0.044069
CISH	1.092242086	0.00024	0.044069
AL358334.2	0.69015873	0.000242	0.04418
C7orf57	-0.792822169	0.000246	0.04467
AC008592.5	1.202713263	0.000247	0.04467
C5orf67	-1.236911671	0.000249	0.044897
AL138902.1	0.855208589	0.000252	0.045308
GADD45A	1.051744853	0.000255	0.045692
LINC01356	-0.799416538	0.000258	0.045933
TRH	-1.034235468	0.000262	0.046442
KCNA5	-0.490561401	0.000268	0.046961
PRND	-1.40314689	0.000268	0.046961
TRPV4	-0.91819649	0.000268	0.046961
CT69	0.804341352	0.000272	0.047452
FAS	**0.595833095**	**0.000274**	**0.047458**
NRARP	-0.440370537	0.000275	0.047458

FNDC7	-0.553653377	0.000277	0.047564
RN7SL141P	0.772141916	0.000278	0.047564
ATP6V0A4	-0.527141477	0.000282	0.048052
MIR7-3HG	-0.730085111	0.00029	0.04906
LCN6	1.351885293	0.00029	0.04906
NAMPT	0.644298803	0.000293	0.049315
PALM2	0.35188116	0.000294	0.049403
AL354928.1	-0.721355282	0.000299	0.049644
PLIN2	0.494412664	0.0003	0.049644
NDRG1	0.328165407	0.0003	0.049644
RFTN1	0.398567279	0.000301	0.049644
HIST1H2APS3	1.520262583	0.000302	0.049644

Bold, DE genes part of the TNF superfamily pathways; LogFC-positive values, upregulated in schizophrenia compared to controls; LogFC-negative values, downregulated in schizophrenia compared to controls.

Table Supplementary 10. Positive correlations between TNFSF receptors and their downstream signaling pathway mRNA markers

TNFSF receptor mRNAs / TNFRSF downstream mRNAs	*DR4/TNFRSF10A*	*FAS/TNFRSF6*	*TWEAKR/ TNFRSF12A*	*TNFR1/ TNFRSF1A*	*TNFR2/ TNFRSF1B*
BAX	*rho*=0.171	*rho*=0.379	*rho*=0.174	*rho*=0.395	*rho*=0.266
BCL2	*rho*=0.375	*rho*=0.651	*rho*=0.166	*rho*=0.672	*rho*=0.292
BID	*rho*=0.305	*rho*=0.134	*rho*=0.364	*rho*=0.211	*rho*=0.135
CASP3	*rho*=0.070	*rho*=0.072	*rho*=-0.186	*rho*=0.131	*rho*=-0.060
CASP9	*rho*=0.106	*rho*=0.307	*rho*=3.02E-13	*rho*=0.203	*rho*=0.133
CASP7	*rho*=0.434	*rho*=0.516	*rho*=-0.272	*rho*=0.458	*rho*=0.386
CASP1	*rho*=0.285	*rho*=0.291	*rho*=0.207	*rho*=0.318	*rho*=0.482
CASP8	*rho*=0.174	*rho*=0.322	*rho*=-0.171	*rho*=0.213	*rho*=0.359
MCL1	*rho*=0.534	*rho*=0.596	*rho*=0.322	*rho*=0.714	*rho*=0.444
TP53	*rho*=0.484	*rho*=0.658	*rho*=0.115	*rho*=0.649	*rho*=0.384

- $p \leq 0.0001$
- $p \leq 0.001$
- $p \leq 0.01$
- $p \leq 0.05$
- *n.s.*

n.s., not significant; underlined, statistical trend.

Table Supplementary 11. Correlations between clinical and dependent variables in the psychiatric groups of the whole mRNA cohort

Demographics	*DR4/TNFRSF10A*	*FAS/ TNFRSF6*	*TWEAKR/ TNFRSF12A*	*TNFR1/ TNFRSF1A*	*TNFR2/ TNFRSF1B*	*GFAP*
Age (years) at onset	*rho*=-0.093, *p*=0.376	***rho*=-0.238, *p*=0.024**	*rho*=-0.038, *p*=0.720	*rho*=0.006, *p*=0.957	*rho*=-0.136, *p*=0.201	***rho*=-0.229, *p*=0.045**
Duration of illness (years)	*rho*=0.114, *p*=0.279	***r*=0.335, *p*=0.001**	*rho*=0.080, *p*=0.446	*rho*=-0.039, *p*=0.712	*rho*=0.155, *p*=0.143	*rho*=0.188, *p*=0.077
Lifetime chlorpromazine equivalent dose (mg)	***rho*=0.308, *p*=0.004**	***rho*=0.449, *p*=1.6E-4**	*rho*=0.189, *p*=0.080	*rho*=0.068, *p*=0.539	***rho*=0.265, *p*=0.014**	***rho*=0.288, *p*=0.012**

PMI, post-mortem interval; *RIN*, RNA integrity number. Bold, statistical significance. Underlined, statistical trend.

Table Supplementary 12. Correlations between clinical and dependent variables in the psychiatric groups of the SMRI mRNA cohort

Demographics	*BAX*	*BCL2*	*BID*	*CASP3*	*CASP9*	*CASP1*	*CASP7*	*CASP8*	*MCL1*	*P53*
Age (years) at onset	*rho*=-0.052, *p*=0.682	*rho*=0.034, *p*=0.786	*rho*=-0.185, *p*=0.143	*rho*=0.040, *p*=0.752	*rho*=0.210, *p*=0.090	***rho*=-0.326, *p*=0.009**	*rho*=0.046, *p*=0.721	<u>*rho*=-0.247, *p*=0.051</u>	*rho*=-0.213, *p*=0.086	*rho*=-0.204, *p*=0.109
Duration of illness (years)	*r*=0.090, *p*=0.478	*rho*=0.073, *p*=0.563	*rho*=0.002, *p*=0.986	***r*=-0.275, *p*=0.027**	*r*=-0.134, *p*=0.283	*r*=0.222, *p*=0.075	*rho*=0.220, *p*=0.080	***rho*=0.326, *p*=0.009**	*r*=0.037, *p*=0.769	*r*=0.198, *p*=0.119
Lifetime chlorpromazine equivalent dose (mg)	*rho*=-0.096, *p*=0.450	*rho*=0.201, *p*=0.109	*rho*=0.071, *p*=0.571	*rho*=-0.148, *p*=0.238	*rho*=-0.057, *p*=0.647	*rho*=0.230, *p*=0.065	***rho*=0.448, *p*<0.001**	***rho*=0.484, *p*<0.001**	***rho*=0.266, *p*=0.031**	***rho*=0.331, *p*=0.008**

PMI, post-mortem interval; *RIN*, RNA integrity number. Bold, statistical significance. Underlined, statistical trend.

Figures Supplementary

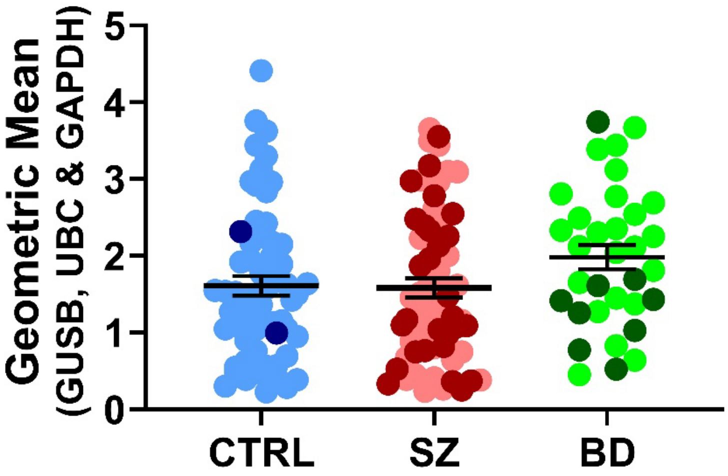

Figure Supplementary 1. Geometric mean expression of the housekeeper genes. Geometric mean expression of the housekeeper genes *GAPDH*, *GUSB*, and *UBC*. *CTRL*, control; *SZ*, schizophrenia; *BD*, bipolar disorder. Darker points are high-inflammation cases. Data are mean ± SEM. Data points are individual cases.

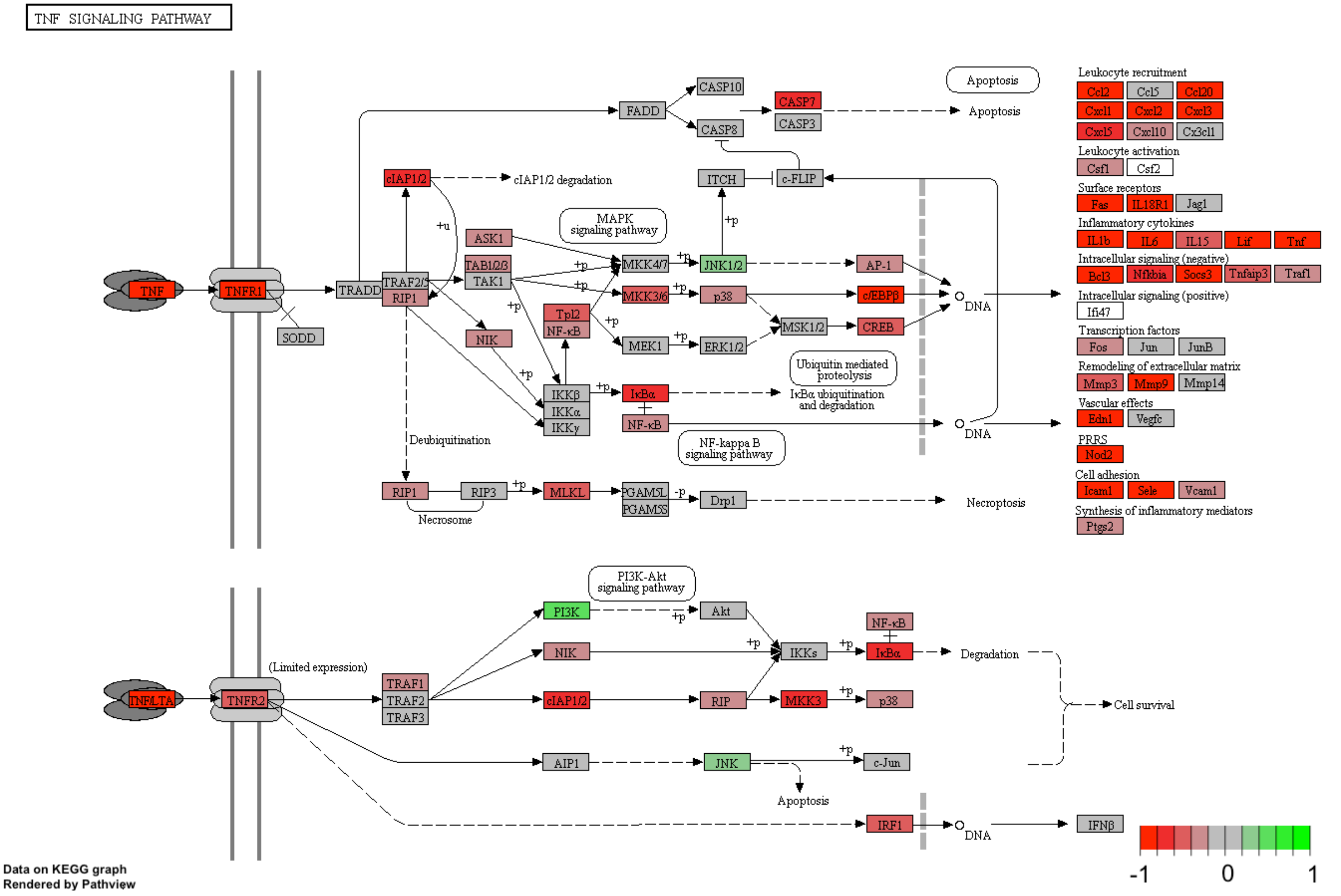

Figure Supplementary 2. Bulk RNA-seq results of the TNF pathway. Genes labeled red indicate being significantly increased, and genes labeled green indicate being significantly decreased in high-inflammation schizophrenia compared to low-inflammation control. Dark colours represent a higher magnitude of change.

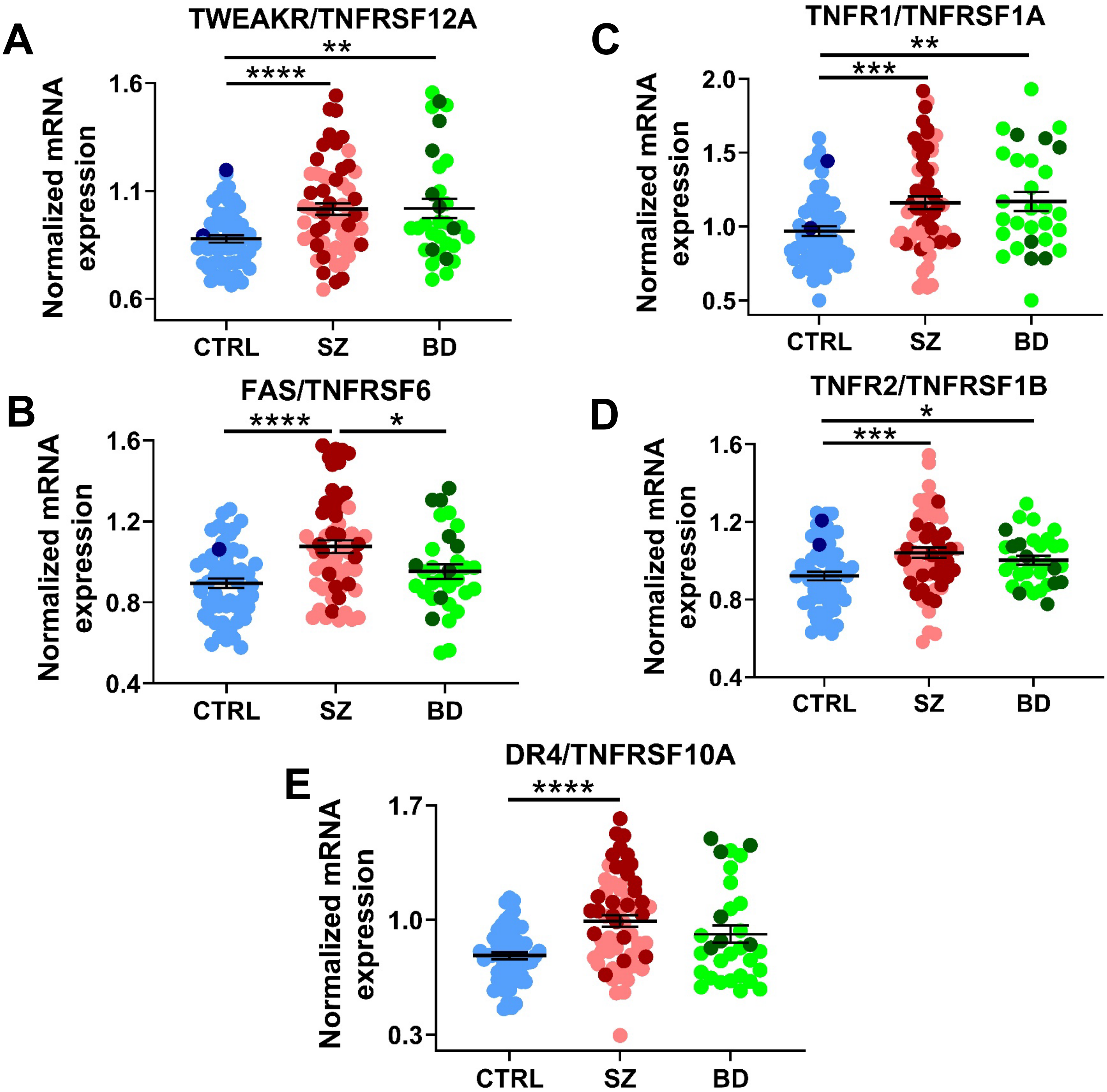

Figure Supplementary 3. mRNA expression of the TNFSF receptors by diagnosis. Normalized mRNA expression levels of the TNFRSF markers TWEAKR (**A**), FAS (**B**), TNFR1 (**C**), TNFR2 (**D**), and DR4 (**E**). CTRL, control; SZ, schizophrenia; BD, bipolar disorder. Darker points are high-inflammation cases. Data are mean ± SEM. Data points are individual cases. *p≤ 0.05, **p≤ 0.01, ***p≤ 0.001, ****p≤ 0.0001.

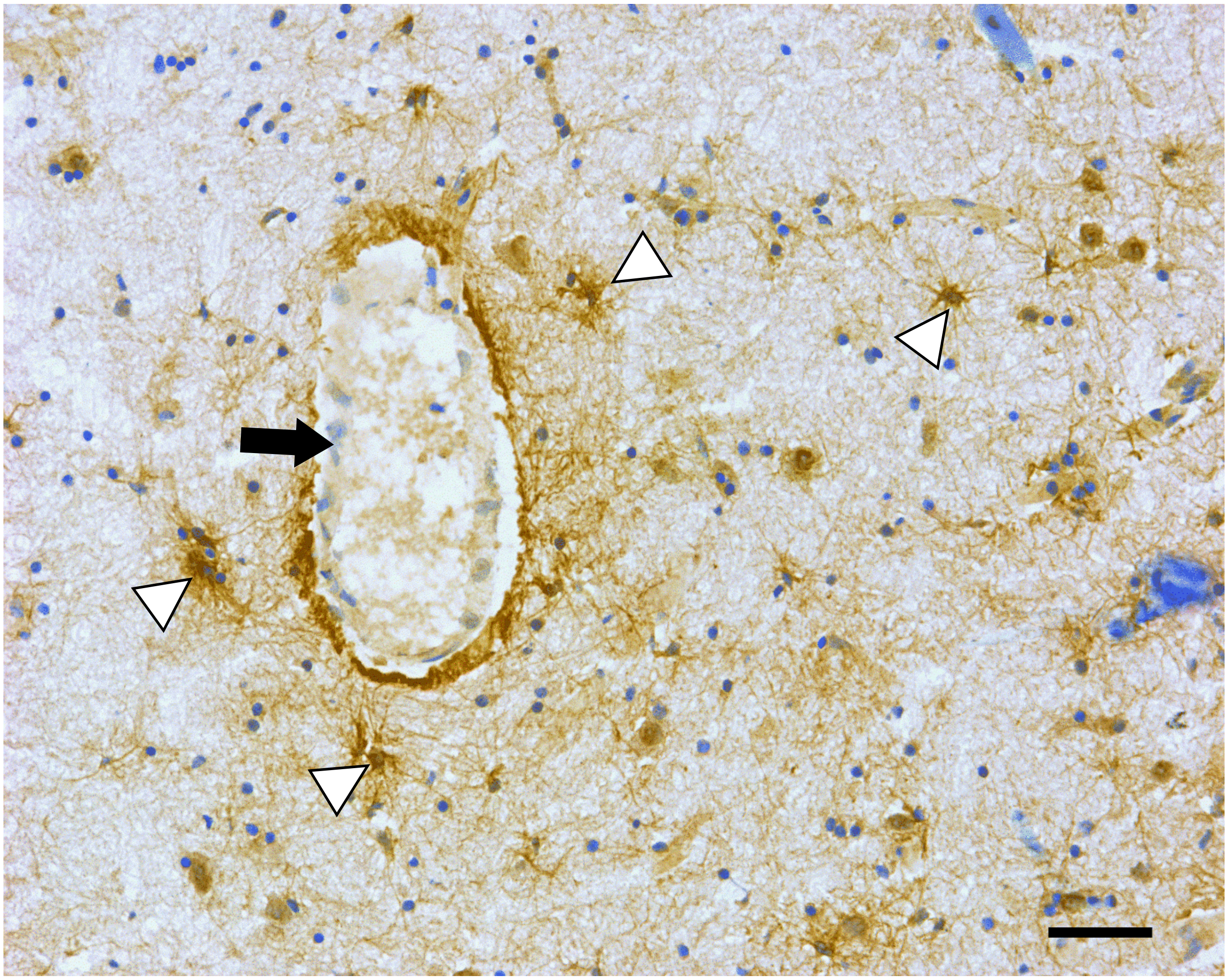

Figure Supplementary 4. Ramified-like FAS+ cells in the dorsal midbrain. Scale bar= 50 µm. White arrowheads, ramified-like FAS+ cells; Black arrow, blood vessel.

References Supplementary

1. Purves-Tyson T, Weber - Stadlbauer U, Richetto J, Rothmond D, Labouesse M, Polesel M, et al. (2019): Increased levels of midbrain immune-related transcripts in schizophrenia and in murine offspring after maternal immune activation. *Molecular Psychiatry*.1-15.
2. Zhu Y, Owens SJ, Murphy CE, Ajulu K, Rothmond D, Purves-Tyson T, et al. (2022): Inflammation-related transcripts define "high" and "low" subgroups of individuals with schizophrenia and bipolar disorder in the midbrain. *Brain, Behavior, and Immunity*.
3. Weickert CS, Sheedy D, Rothmond DA, Dedova I, Fung S, Garrick T, et al. (2010): Selection of reference gene expression in a schizophrenia brain cohort. *Aust N Z J Psychiatry*. 44:59-70.
4. Puvogel S, Alsema A, Kracht L, Webster MJ, Weickert CS, Sommer IEC, et al. (2022): Single-nucleus RNA sequencing of midbrain blood-brain barrier cells in schizophrenia reveals subtle transcriptional changes with overall preservation of cellular proportions and phenotypes. *Molecular Psychiatry*. 27:4731-4740.
5. Hagihara H, Catts VS, Katayama Y, Shoji H, Takagi T, Huang FL, et al. (2018): Decreased Brain pH as a Shared Endophenotype of Psychiatric Disorders. *Neuropsychopharmacology*. 43:459-468.
6. Erra Díaz F, Dantas E, Geffner J (2018): Unravelling the Interplay between Extracellular Acidosis and Immune Cells. *Mediators Inflamm*. 2018:1218297.
